# Supplementary material for: Selection of reference genes for quantitative real-time PCR analysis in cucumber (Cucumis sativus L.), pumpkin (Cucurbita moschata Duch.) and cucumber–pumpkin grafted plants
Source: PeerJ. 2019 Apr 17;7:e6536. doi: 10.7717/peerj.6536 (PMC6475253; doi:10.7717/peerj.6536)
Supplement: Table S1 — This is description of transcriptiome data of graft union at 0, 3, 6, 9 days after grafting. [file peerj-07-6536-s001.docx]

**Table S1. Summary for the graft union transcriptome**

| **Species** | **No.**  **gene** | **Length of gene**  **(Mb)** | **Average of gene**  **(Kb)** | **No. transcripts** | **Length of**  **Transcripts**  **(Mb)** | **Average of**  **Transcripts**  **(K)** | **No.**  **exon** | **Length of**  **Exon**  **(Mb)** | **Average of**  **exon** |
| --- | --- | --- | --- | --- | --- | --- | --- | --- | --- |
| Cucumber | 20,782 | 86.0 | 4.1 | 32,852 | 154.0 | 4.7 | 200,813 | 59.5 | 296 |
| pumpkin | 27,187 | 119.0 | 4.4 | 47,906 | 204.4 | 4.3 | 288,837 | 84.0 | 290 |
