# Supplementary figures and images for: Selection of reference genes for quantitative real-time PCR analysis in cucumber (Cucumis sativus L.), pumpkin (Cucurbita moschata Duch.) and cucumber–pumpkin grafted plants
Source: PeerJ. 2019 Apr 17;7:e6536. doi: 10.7717/peerj.6536 (PMC6475253; doi:10.7717/peerj.6536)

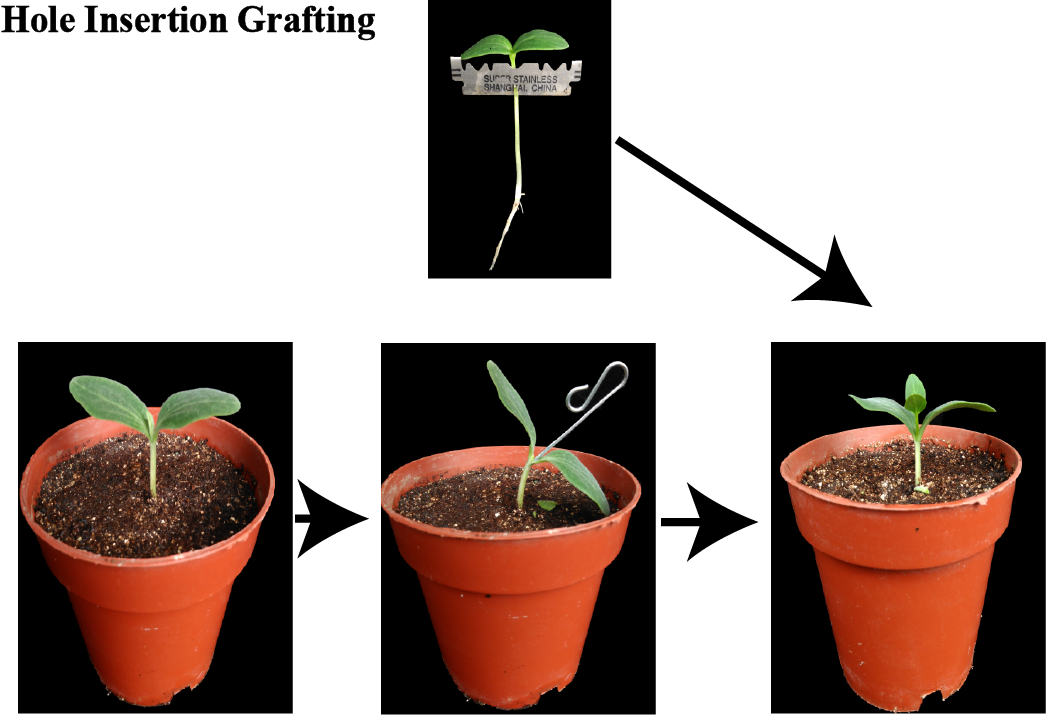

Supplement: Figure S1 — A cucumber cultivar (Zhongnong No.26) was used as the scion, a pumpkin cultivar (Jinxinzhen No.5) was used as the rootstock. The rootstocks were sown 2–3 d earlier than scions (6–7 d after sowing ). When cotyledons of the scion were fully opened and the first true leaf of the rootstock started to develop (9–10 d after sowing) plants grafted as previously described (Mohamed et al., 2014). A hole on the upper portion of the rootstock hypocotyls was made, and then the growing point of the rootstock were removed with a razor blade. The scion was cut on a 30 ° -60 ° on both sides of the hypocotyls, then made the scion insert into the hole made in the rootstock quickly, and the cut surfaces were matched together and held with a grafting clip (Fig. S1). [file peerj-07-6536-s006.png]

**A**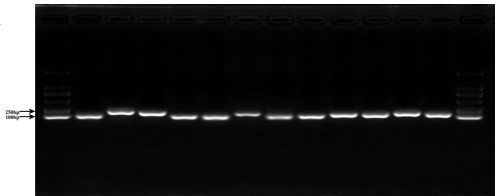**B**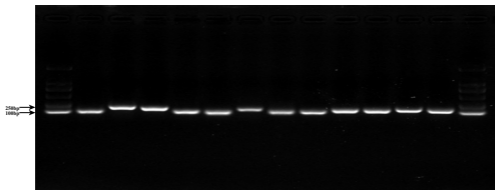**C**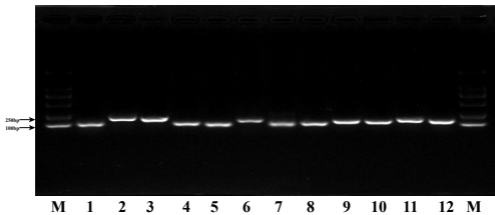

Supplement: Figure S2 — Based on the conserved sequence of these genes between cucumber and pumpkin, primers were designed using Primer Premier 5.0 software with the following parameters: a melting temperature (Tm) of 50–60° C, a primer length of 17–25 bp, and a product size of 70–260 bp (http://www.premierbiosoft.com/) (Table 1). Amplification of a single PCR product in 1% agarose gel electrophoresis. [file peerj-07-6536-s007.pdf]

A

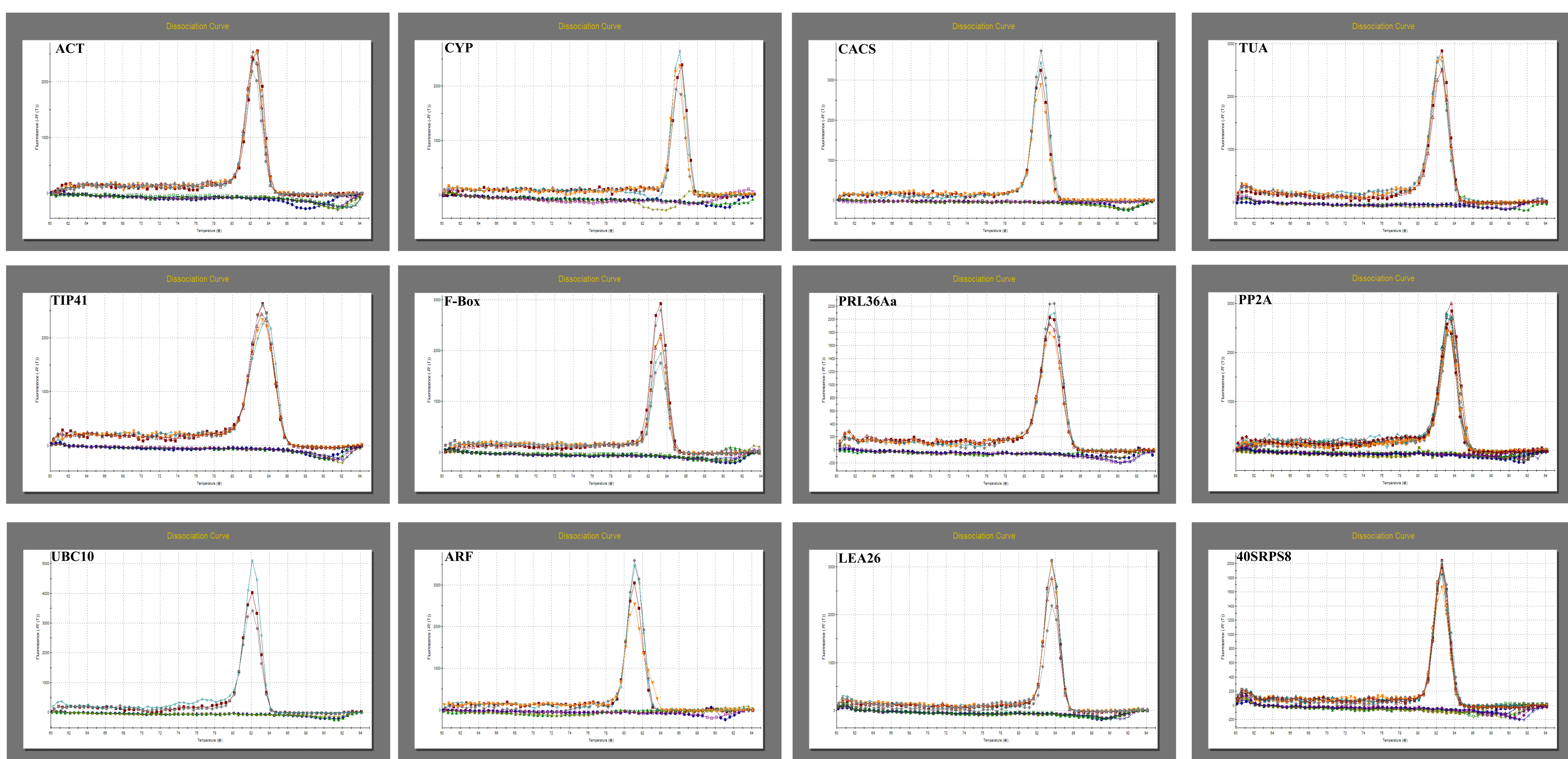

B

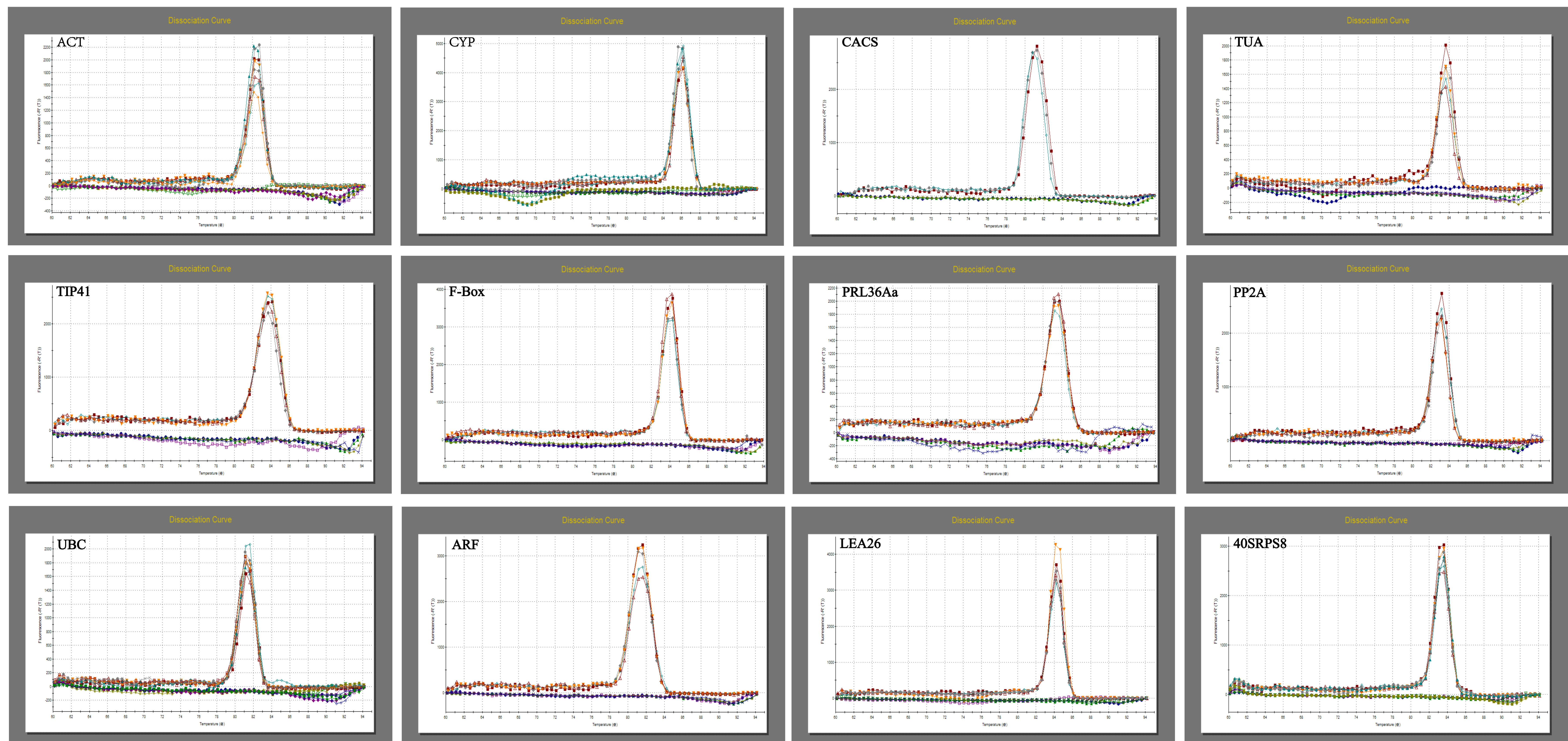

Supplement: Figure S3 — The Fluorescence changes (X-axis) was plotted versus the reaction temperature of qRT-PCR (Y-axis). A single peak of the melting curve in qRT-PCR were used to ensure the specificity of the primers for the candidate reference genes. [file peerj-07-6536-s008.pdf]

A

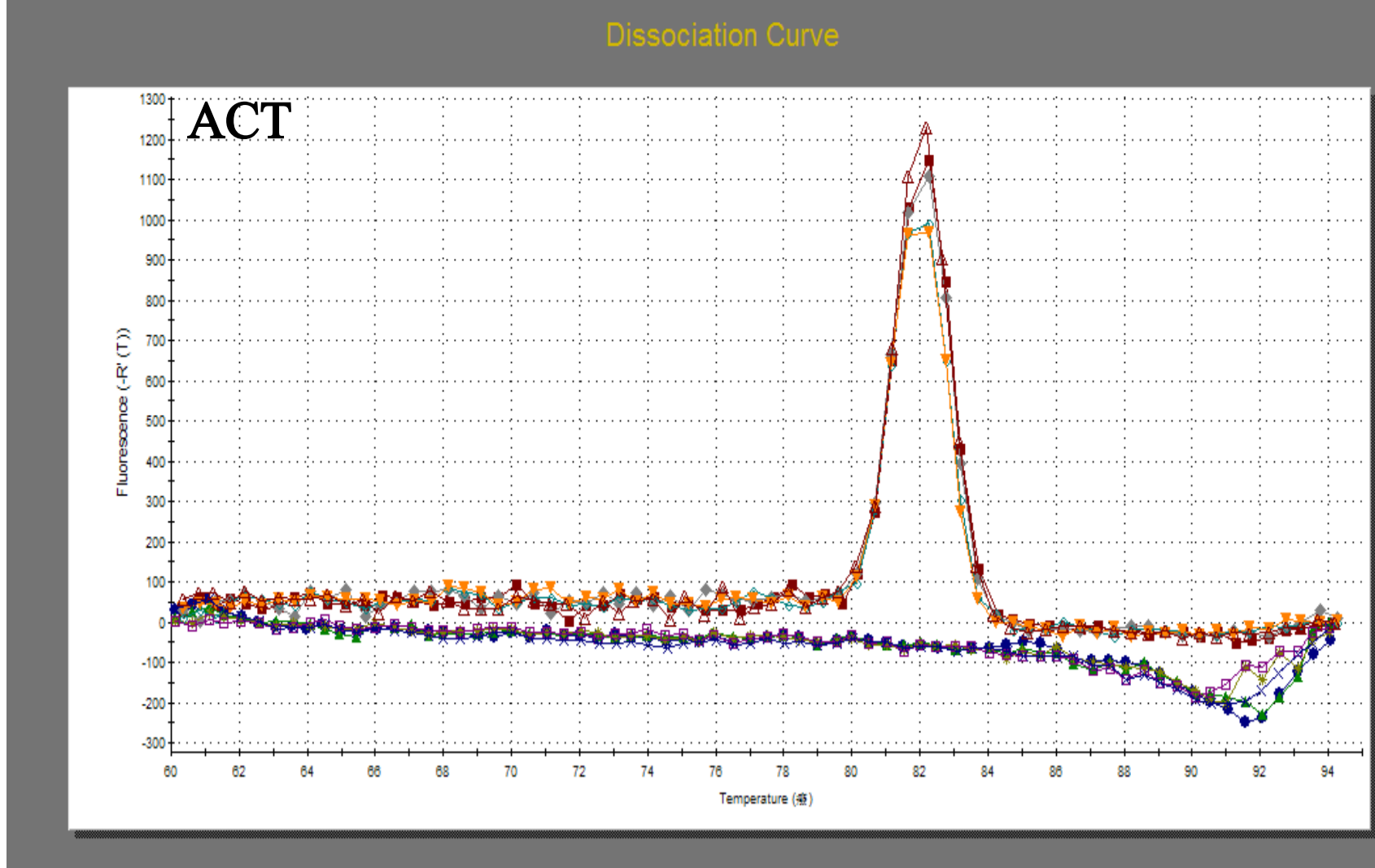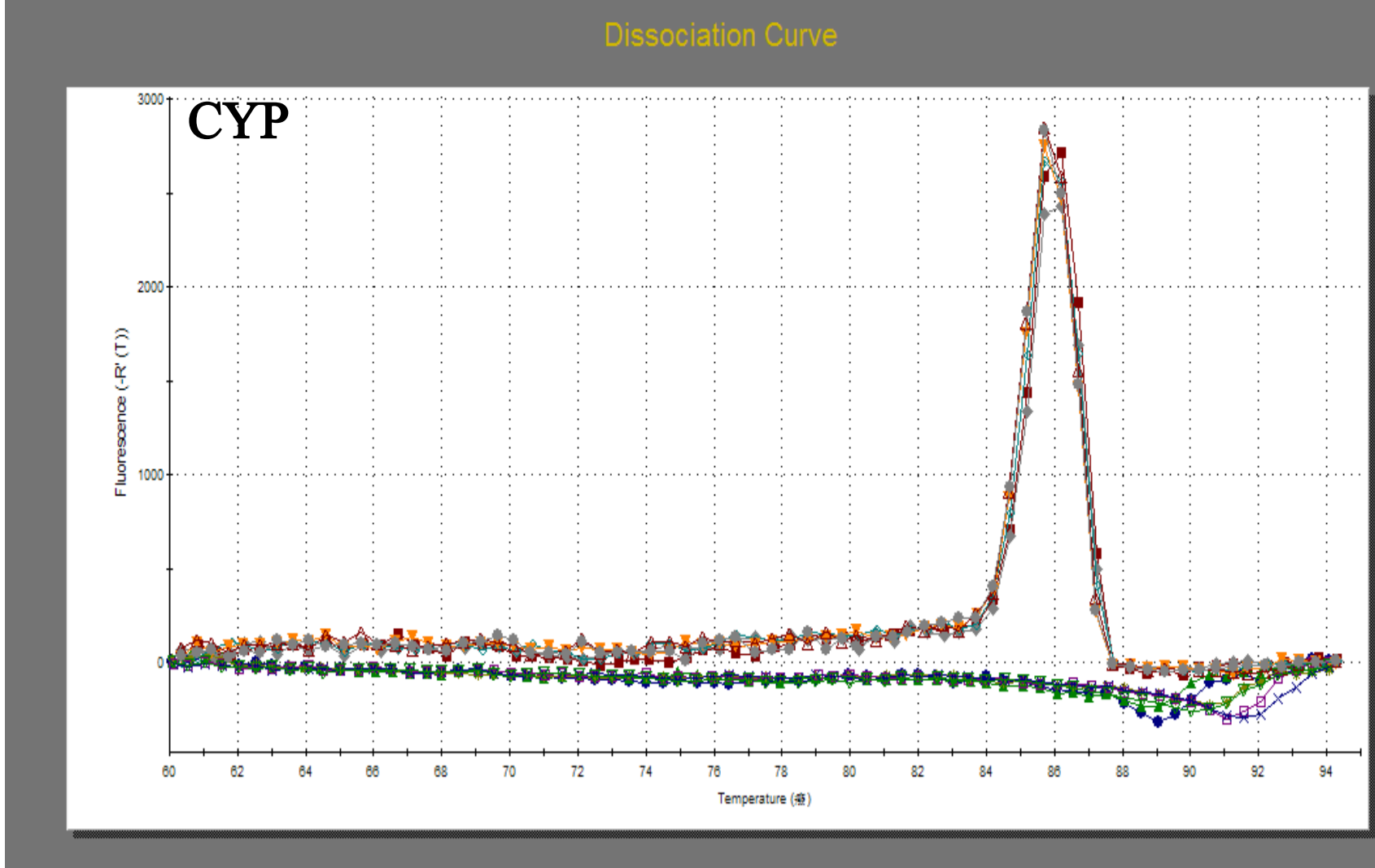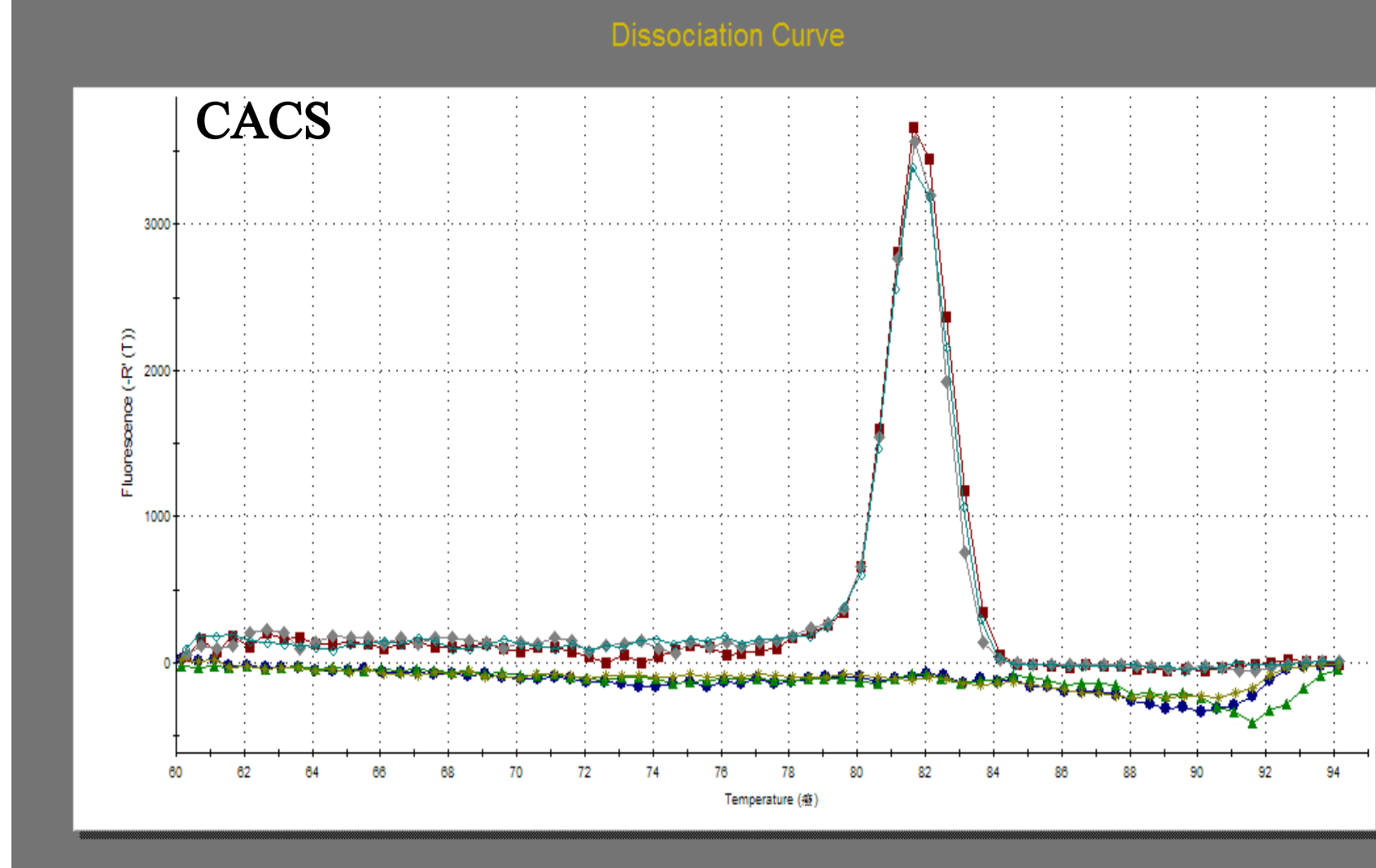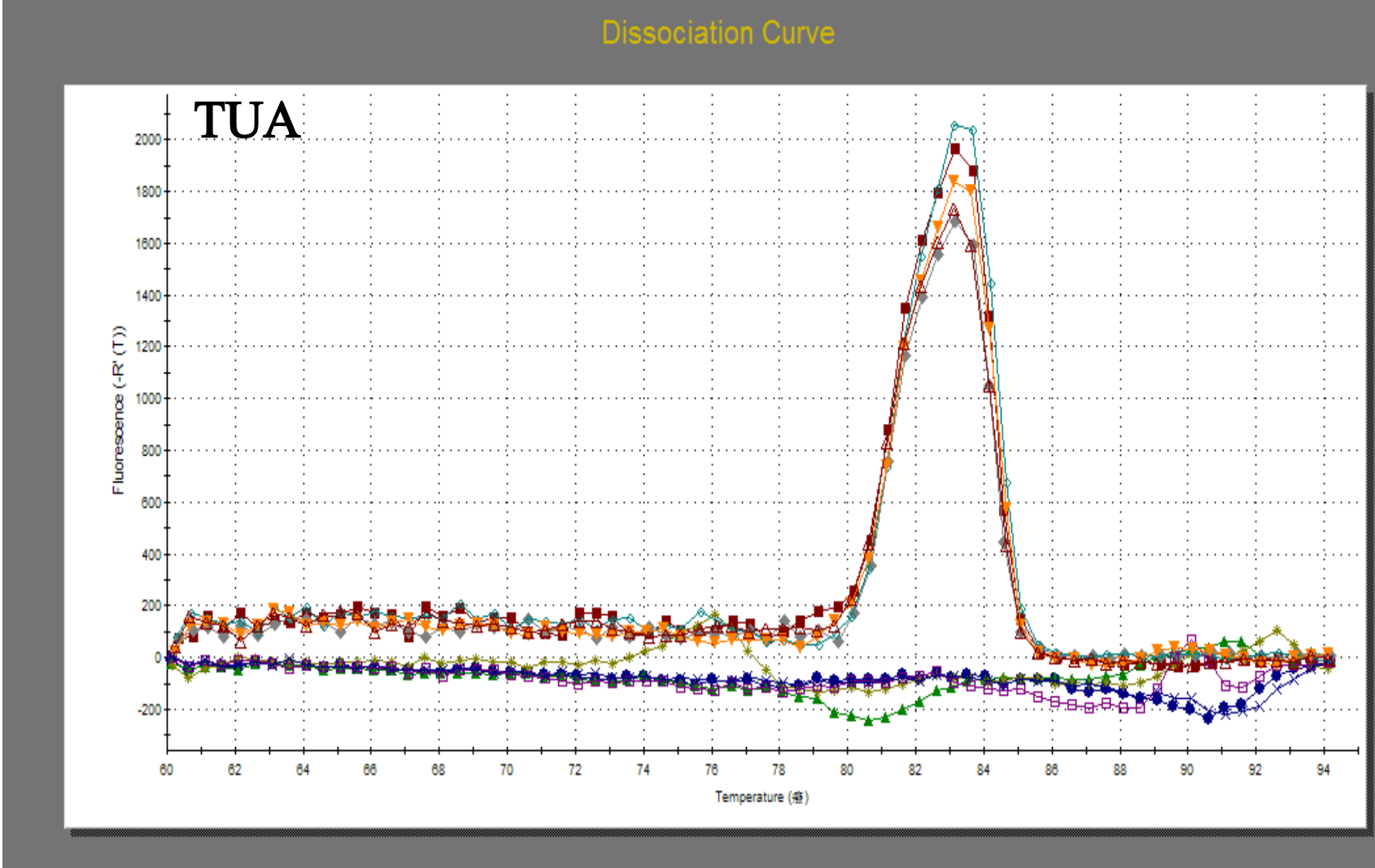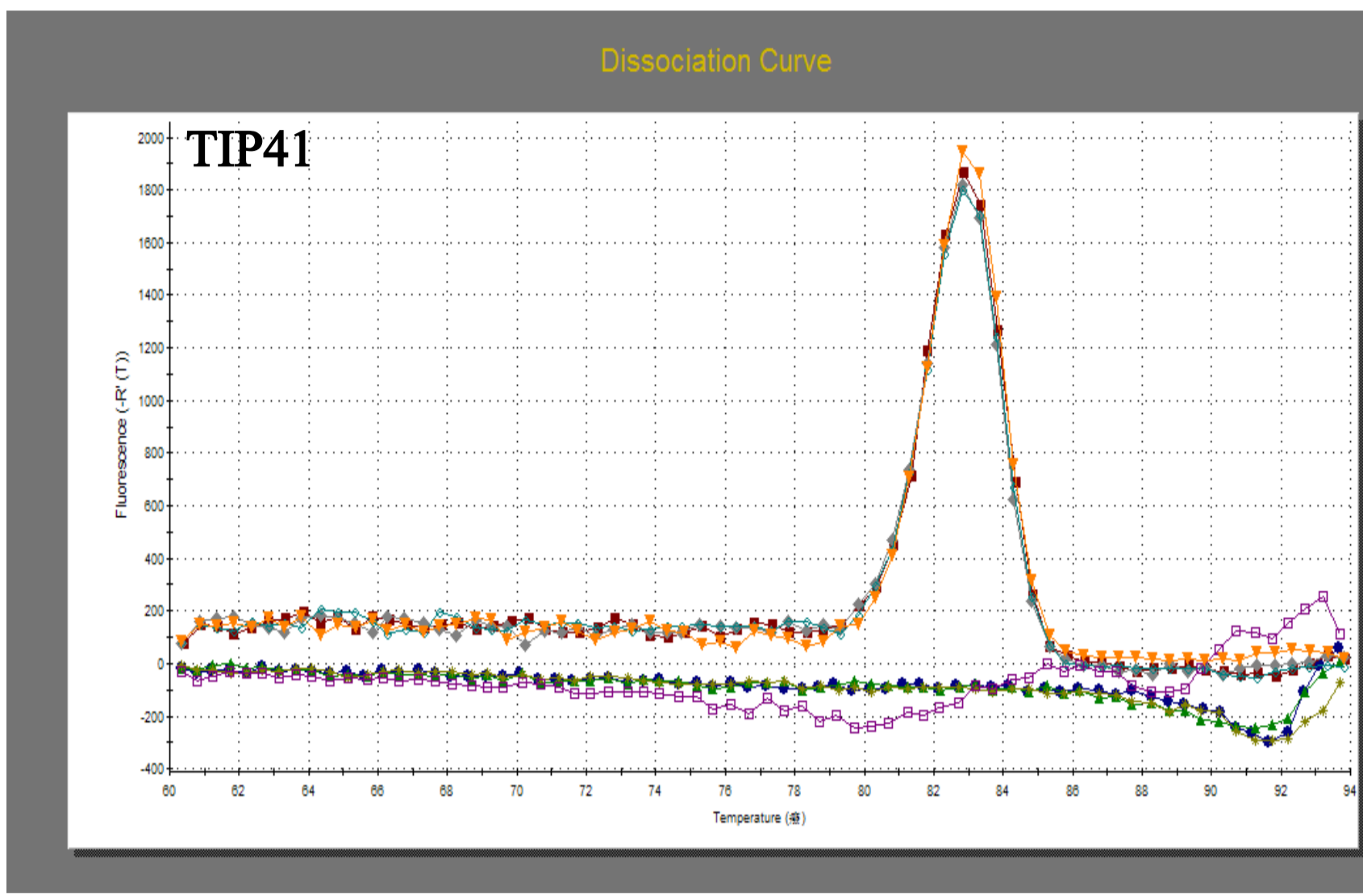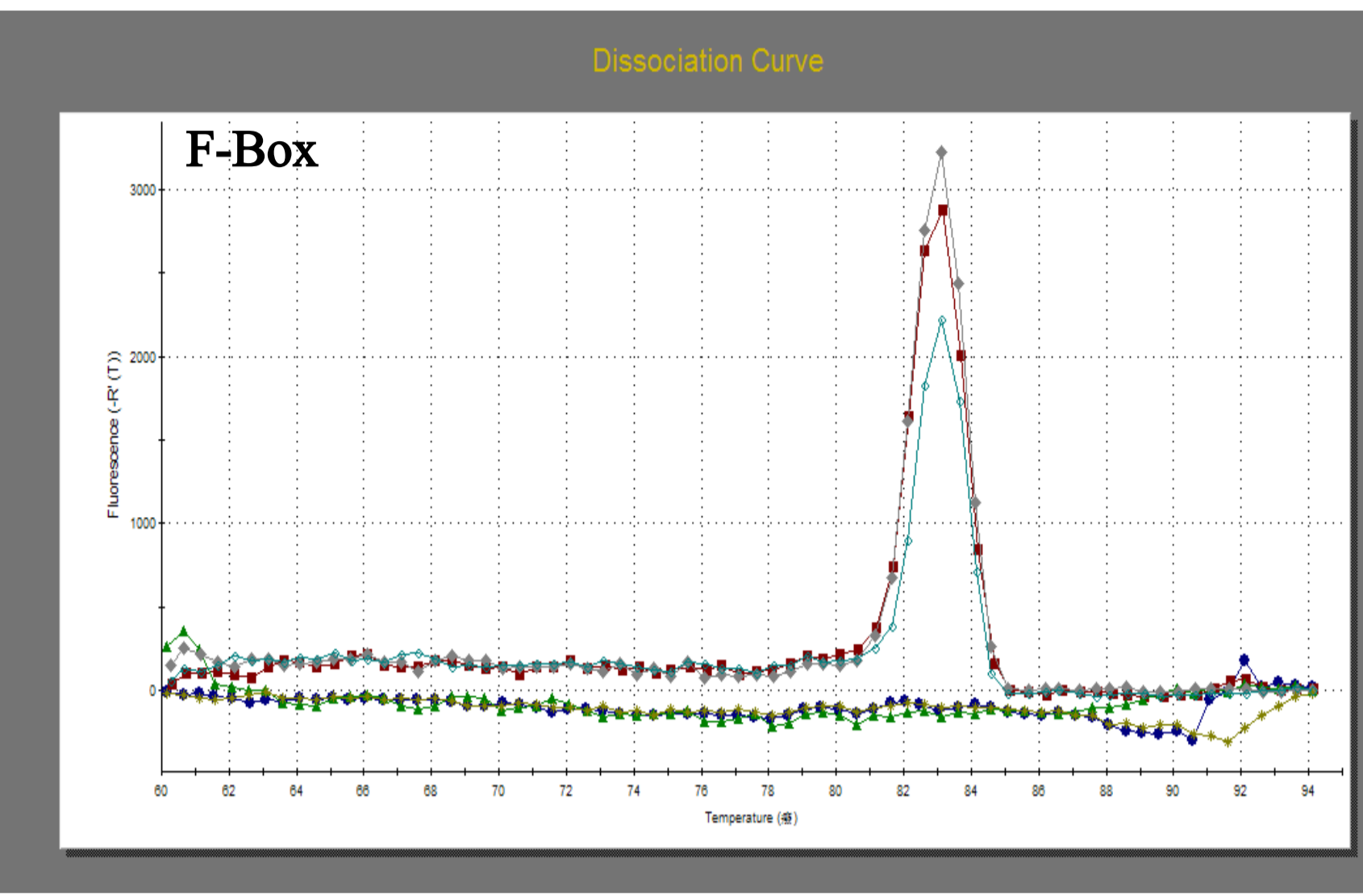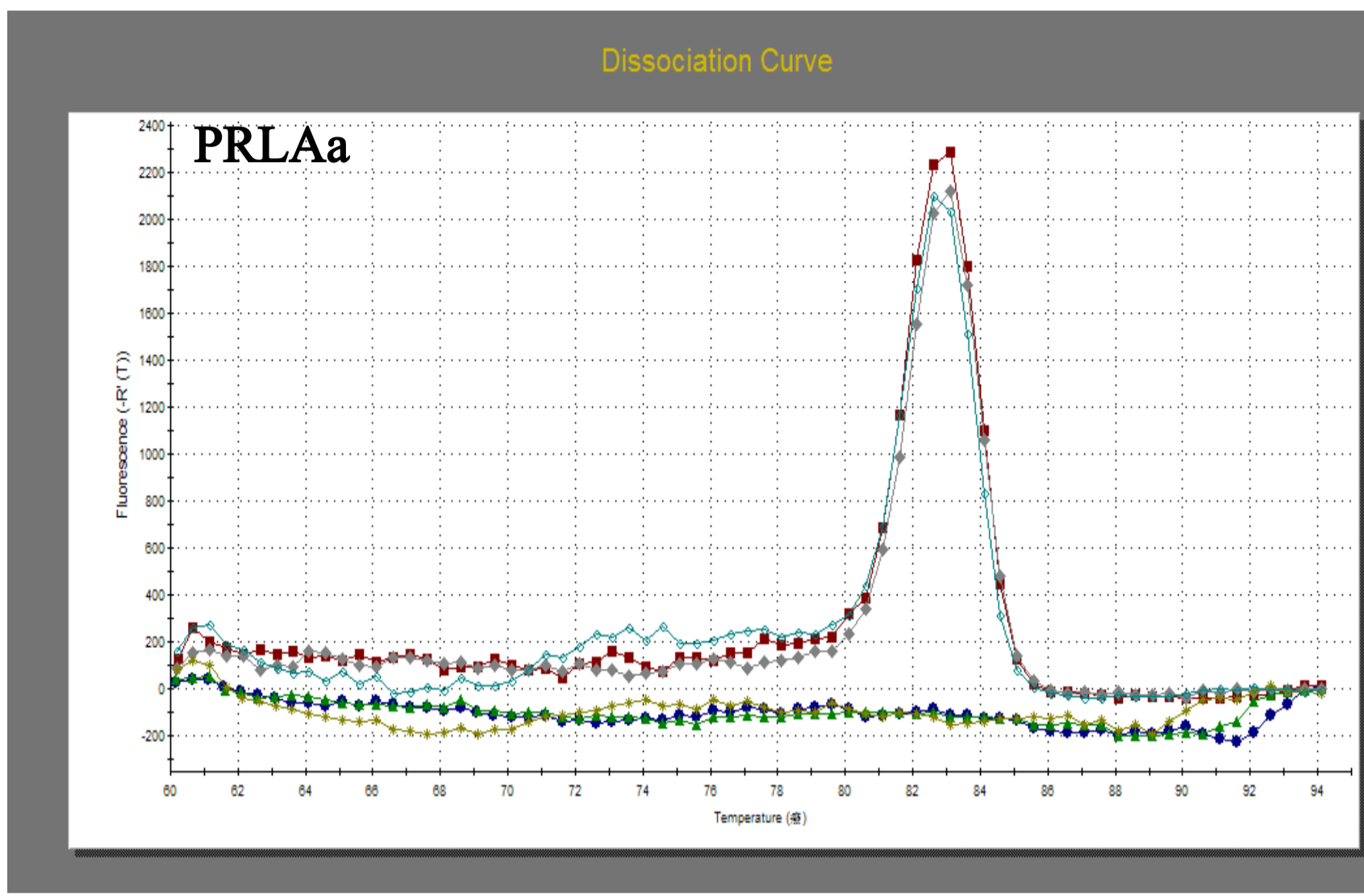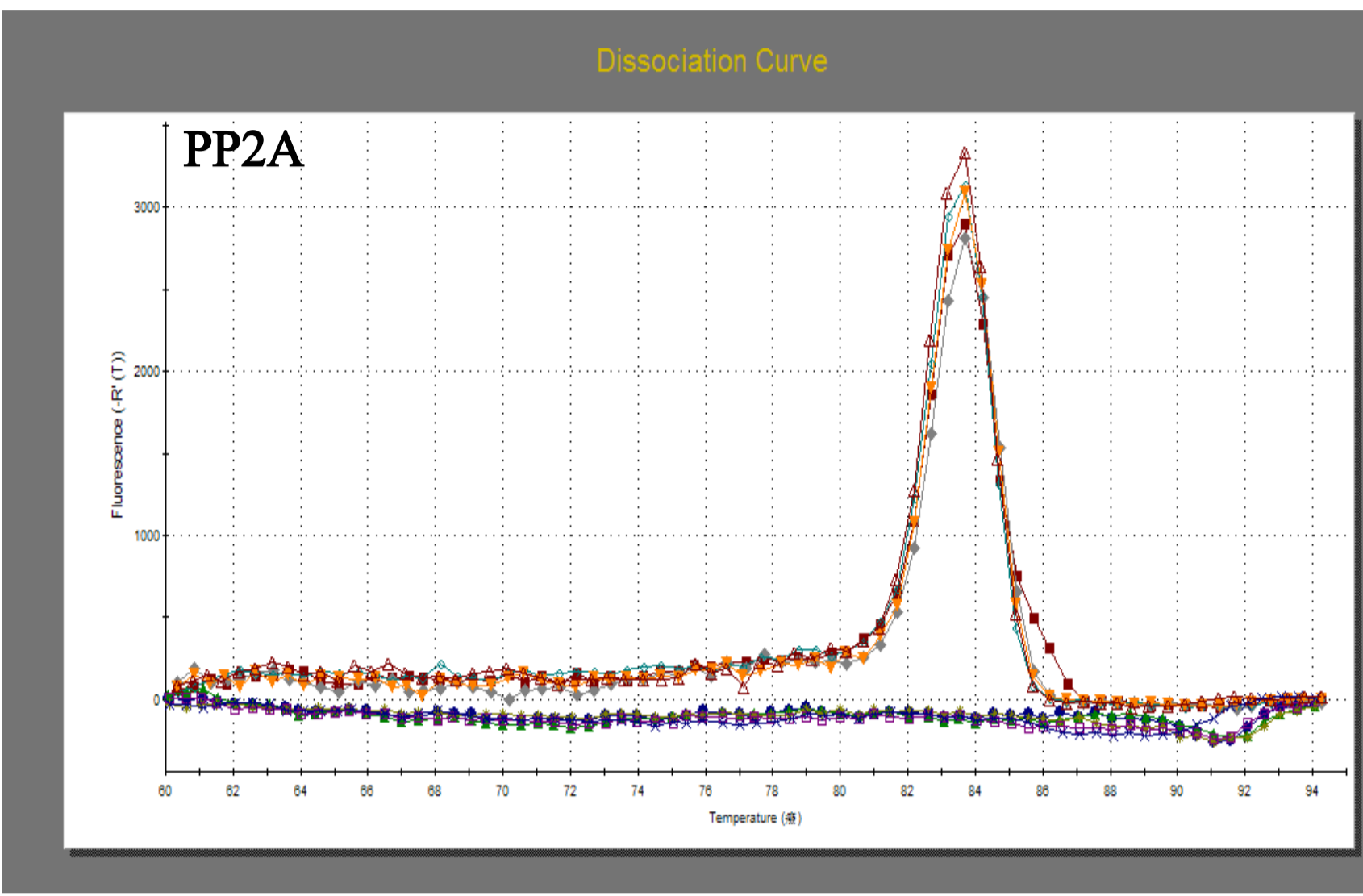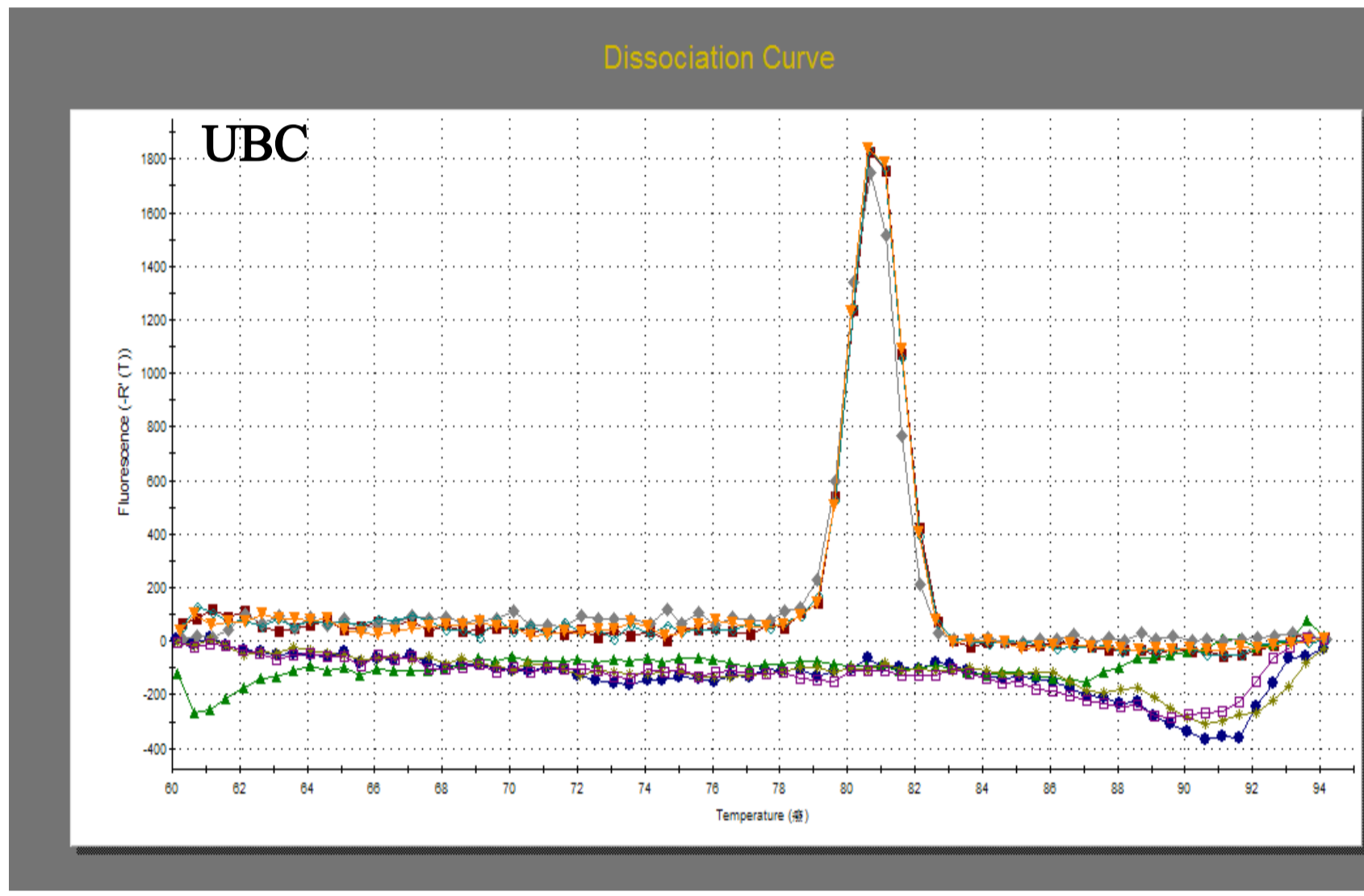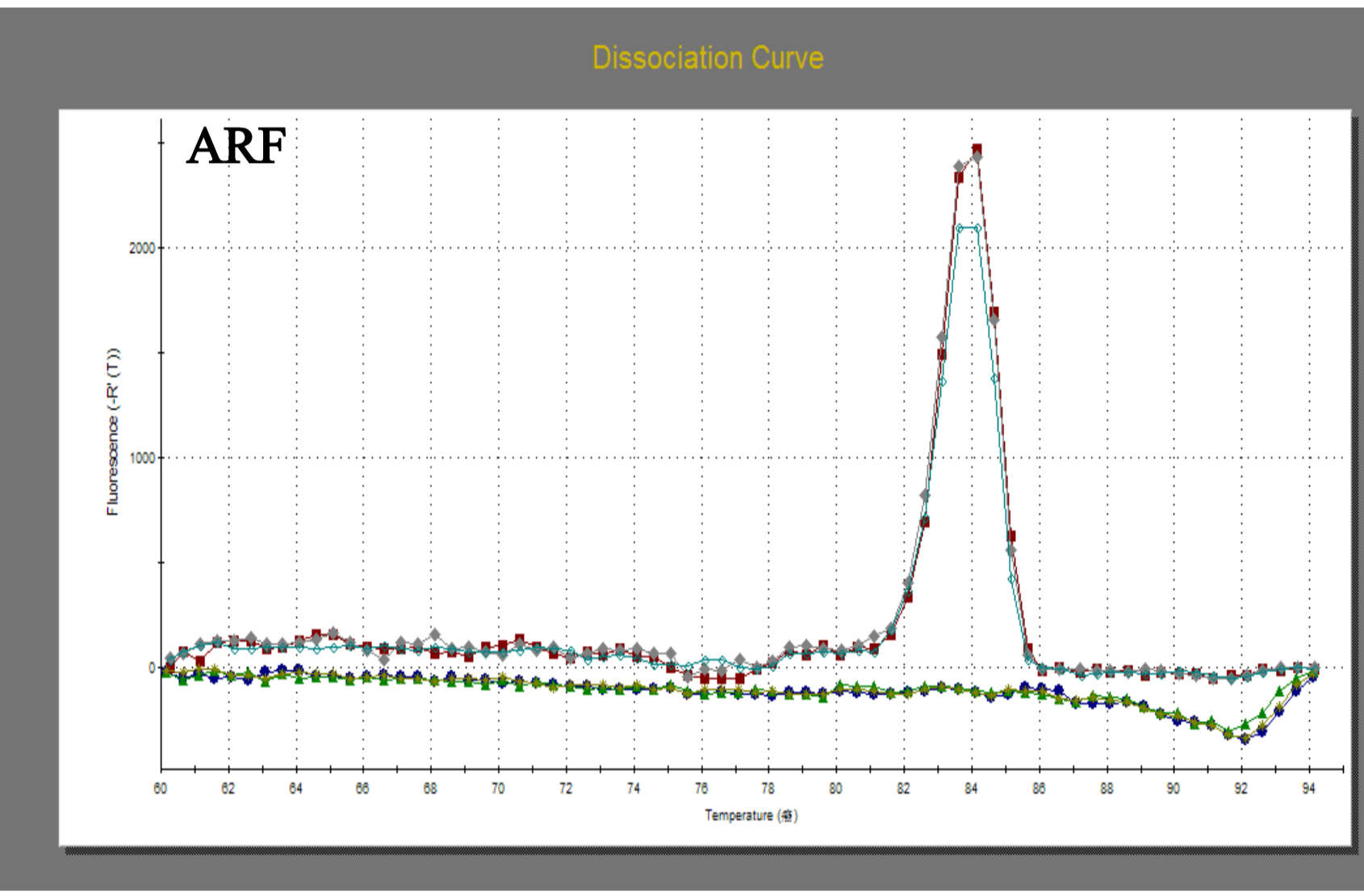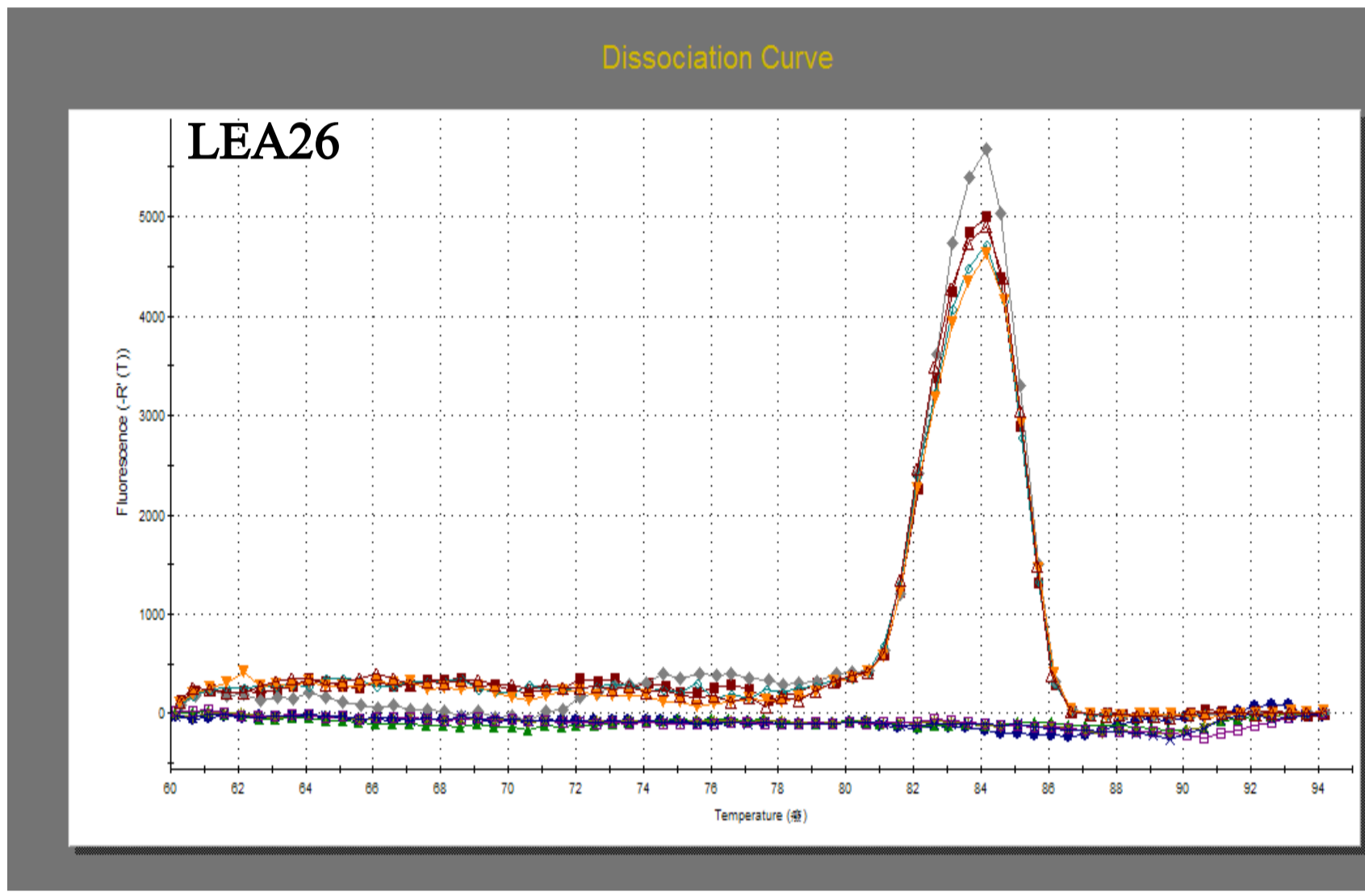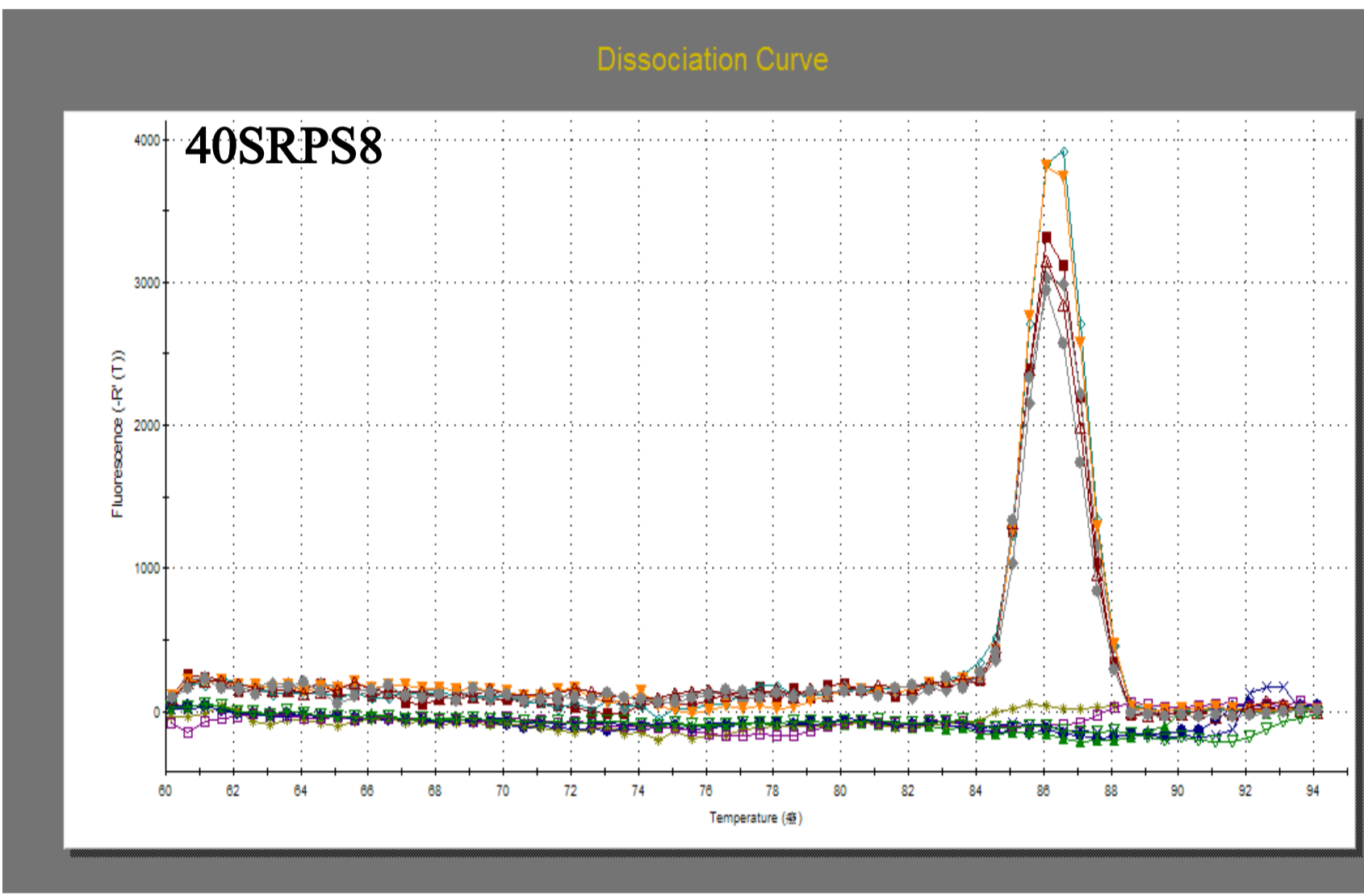

B

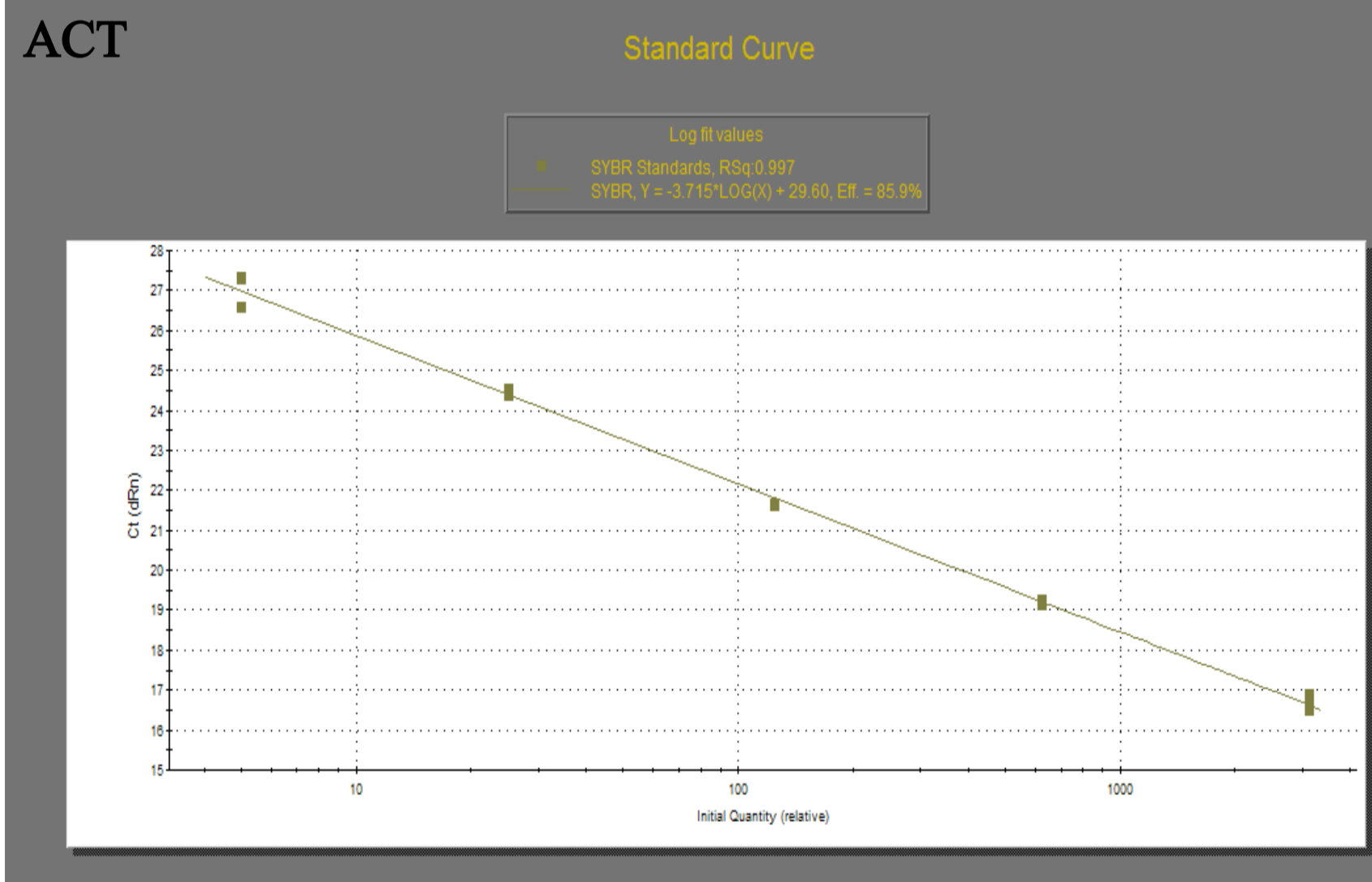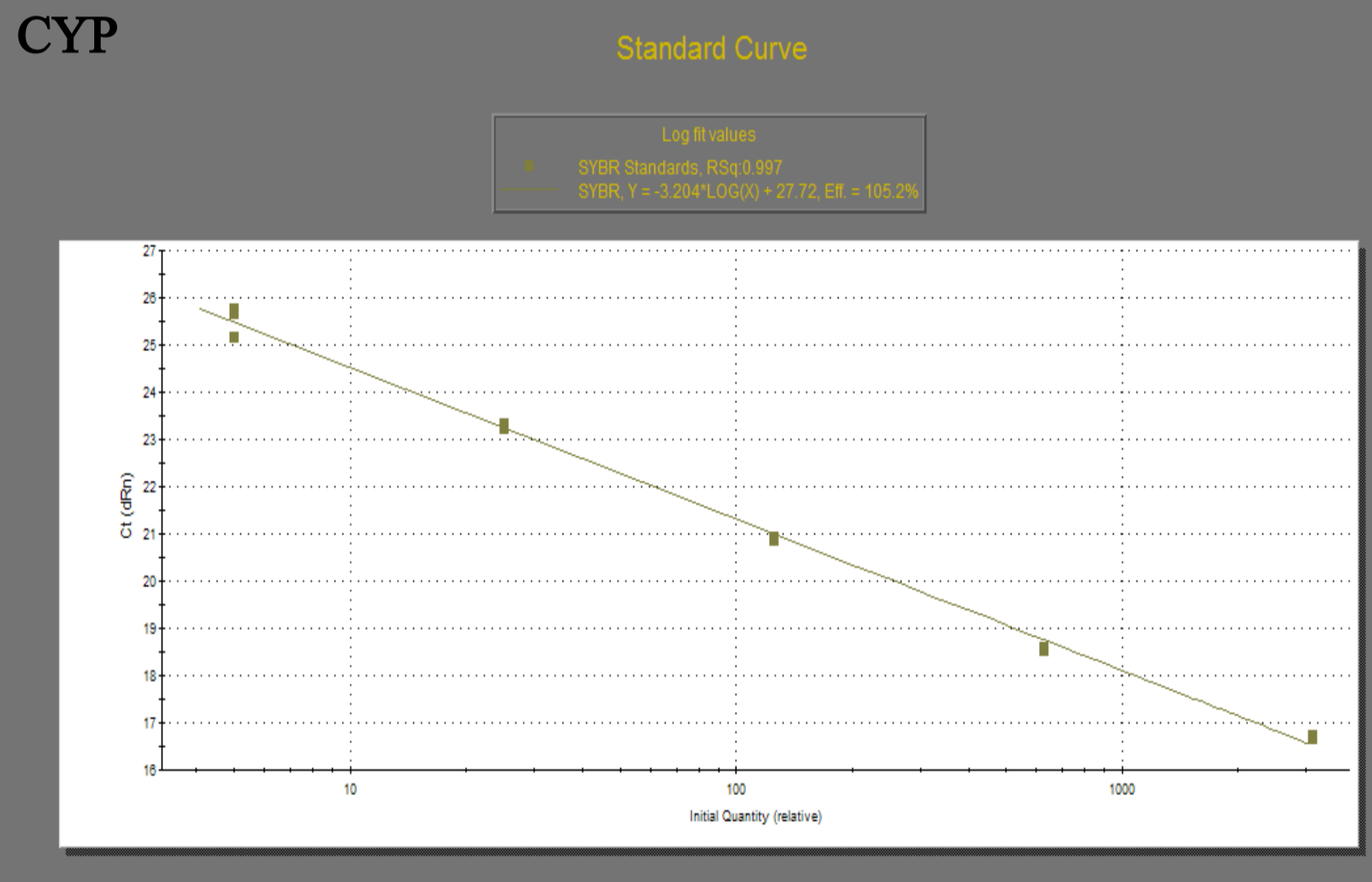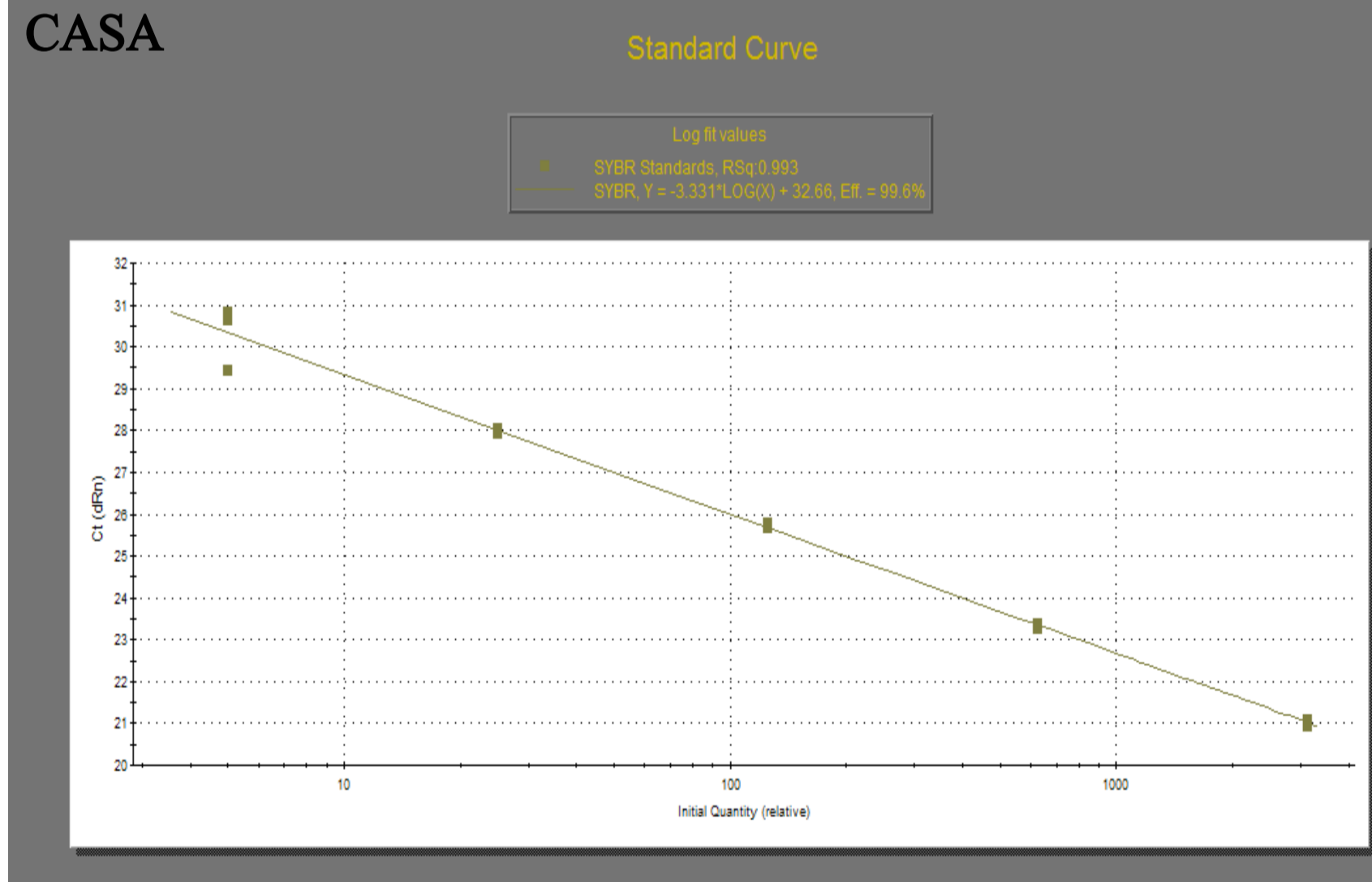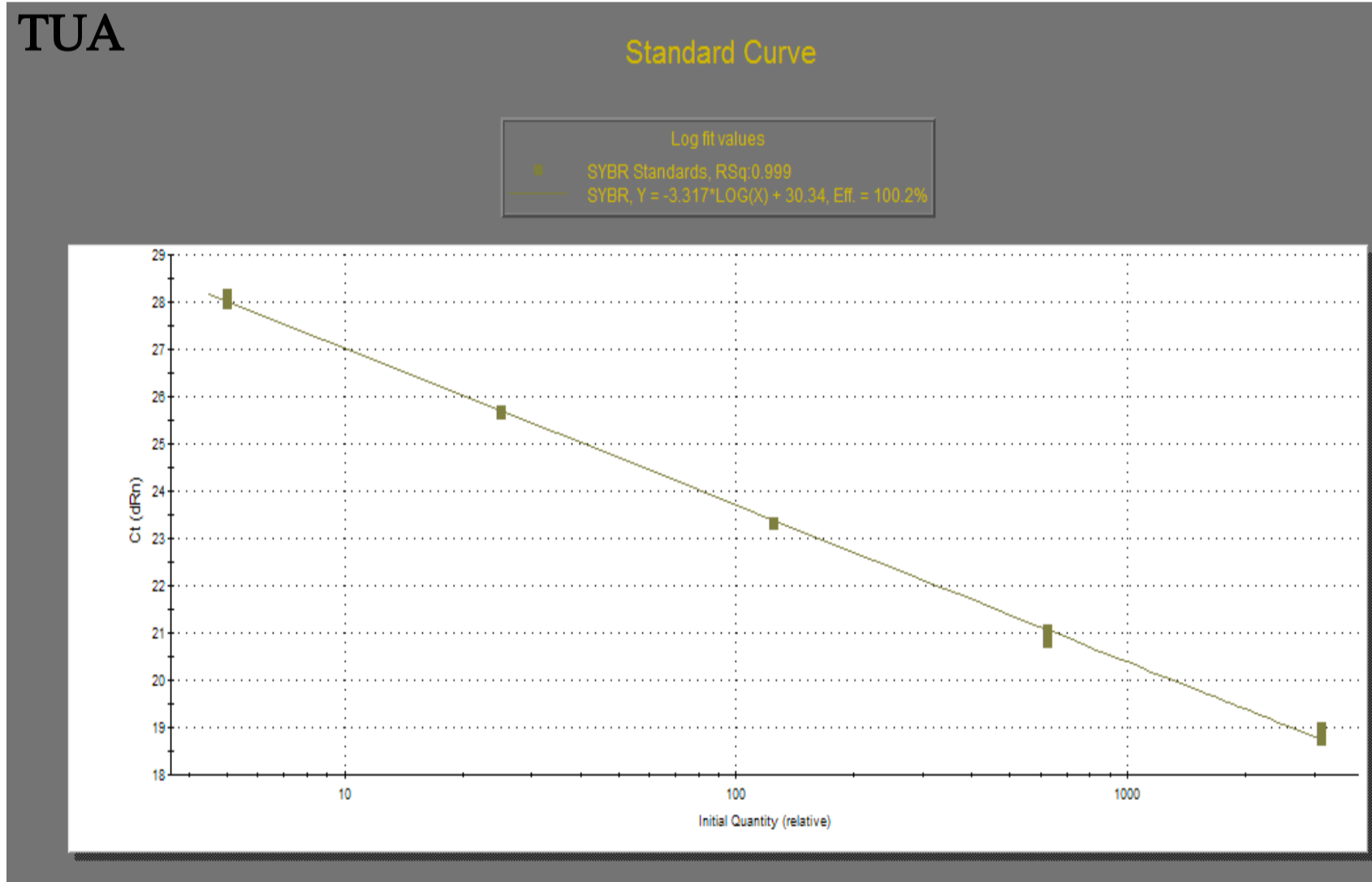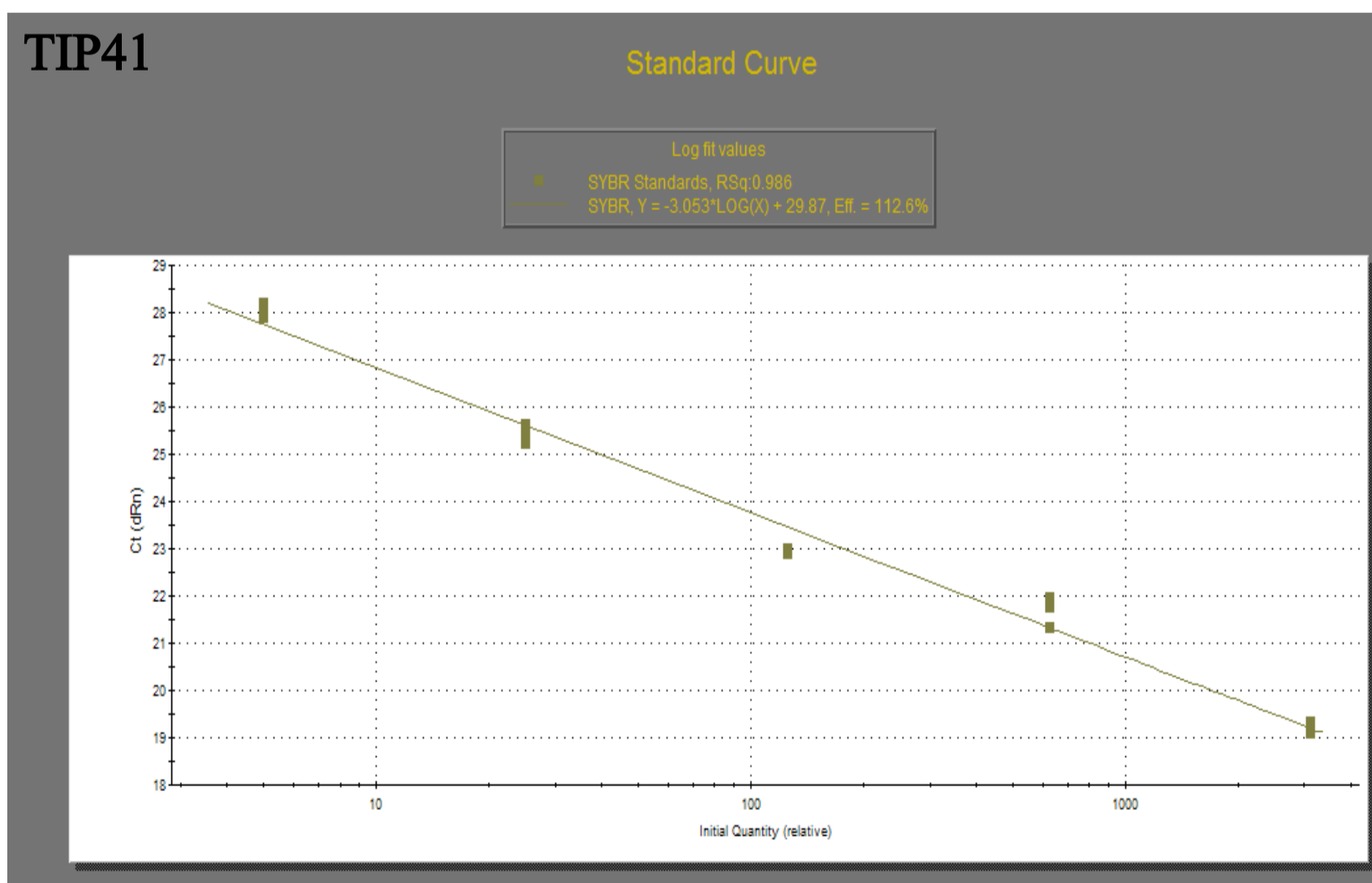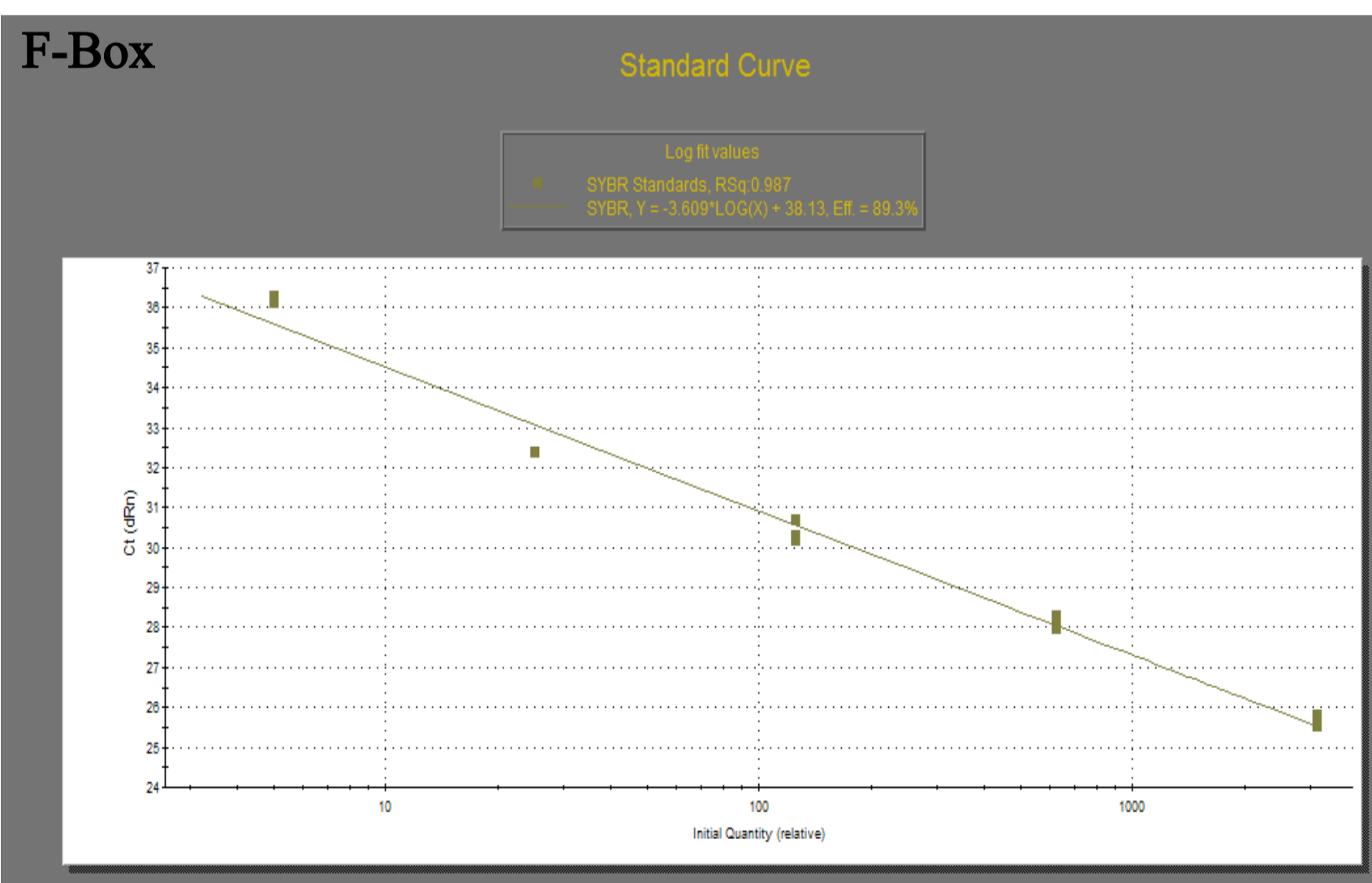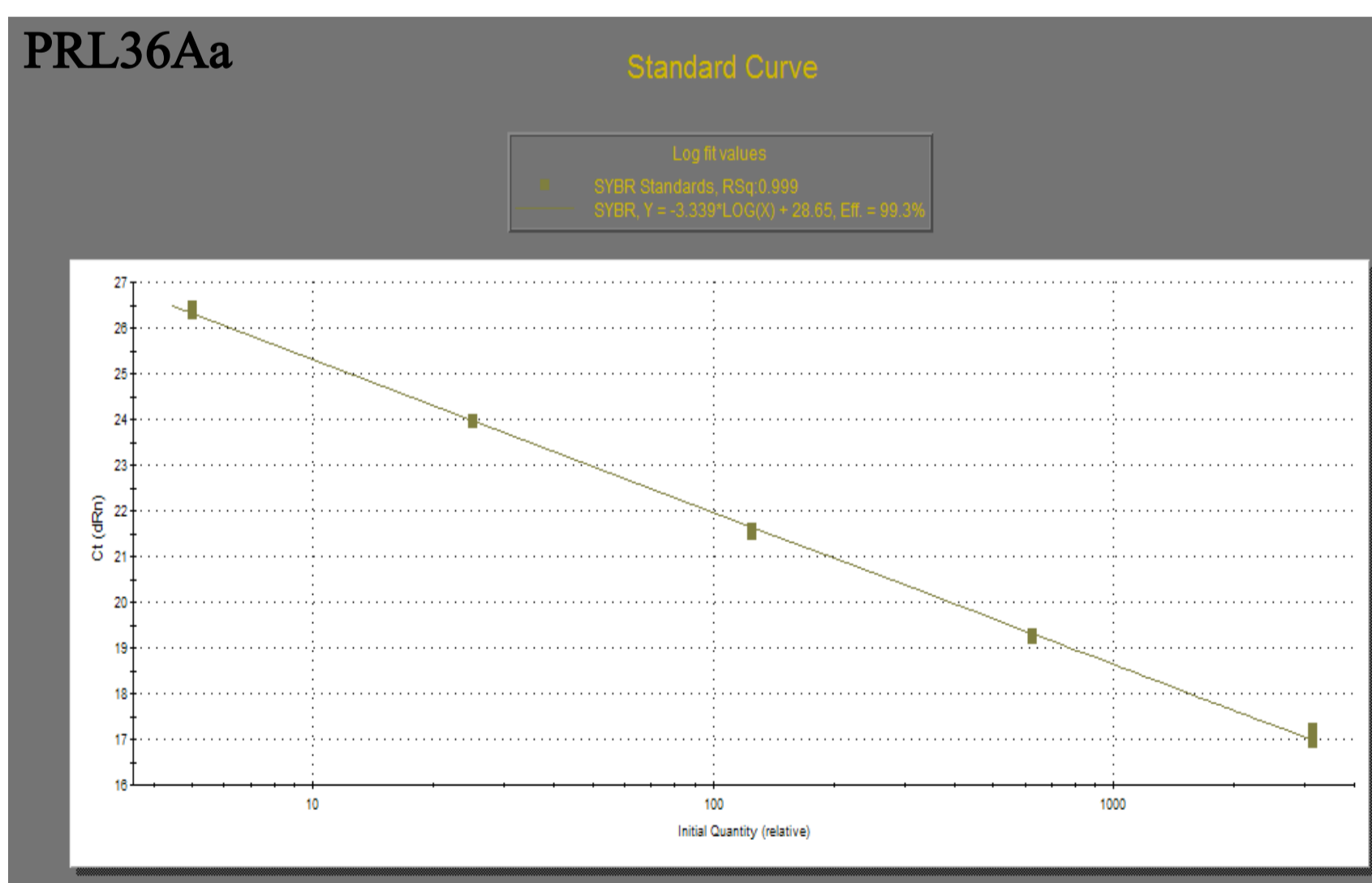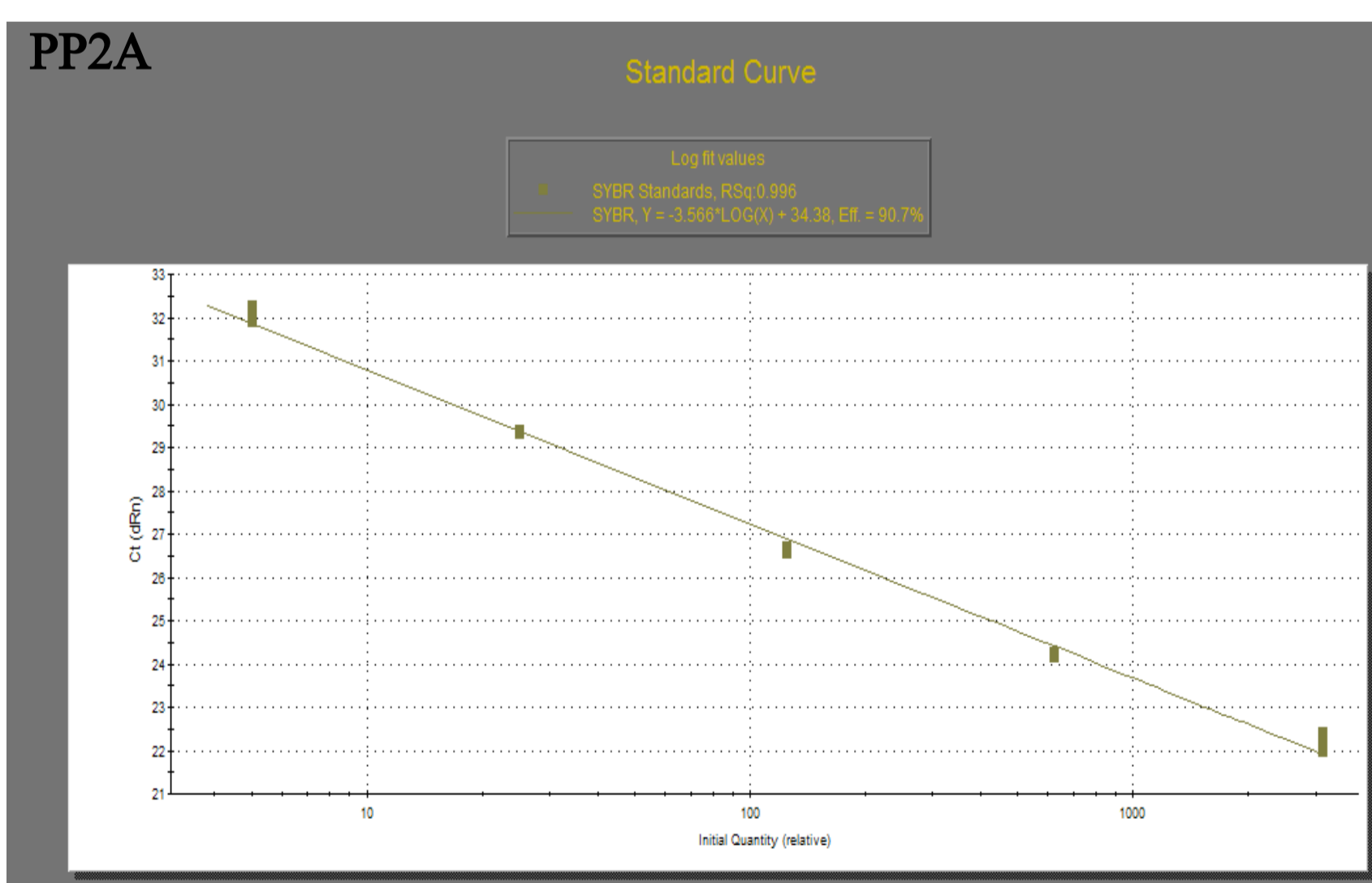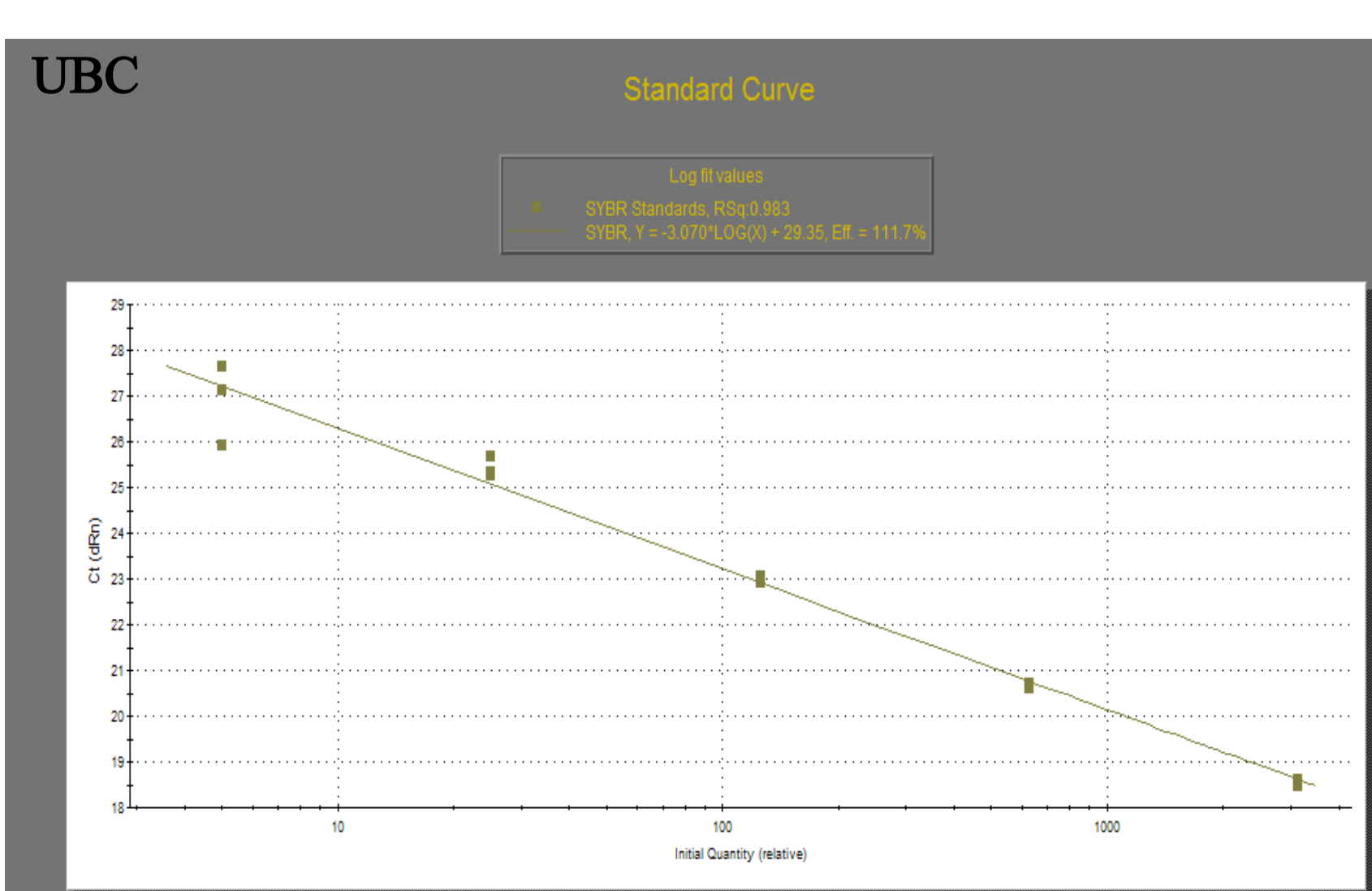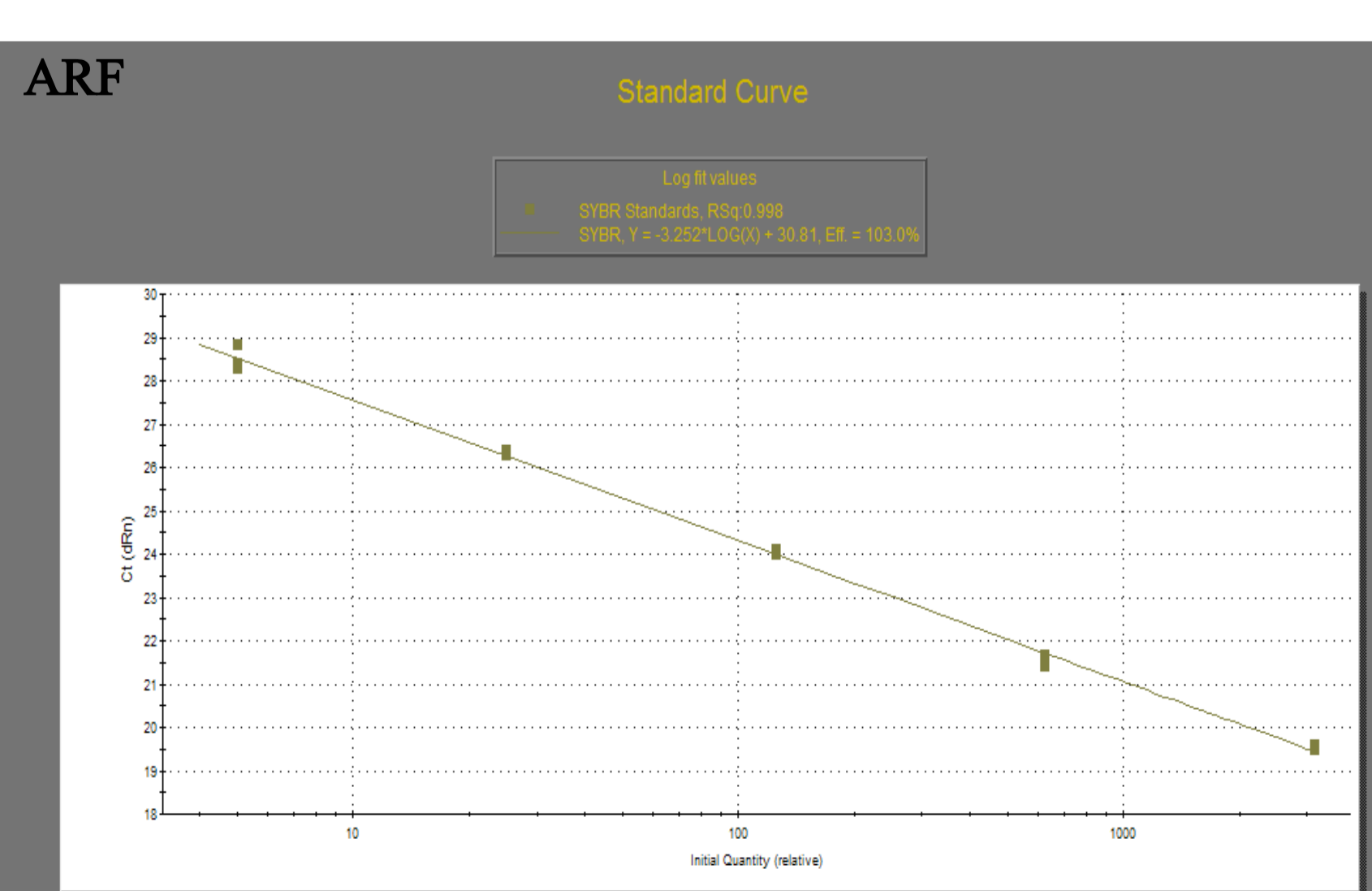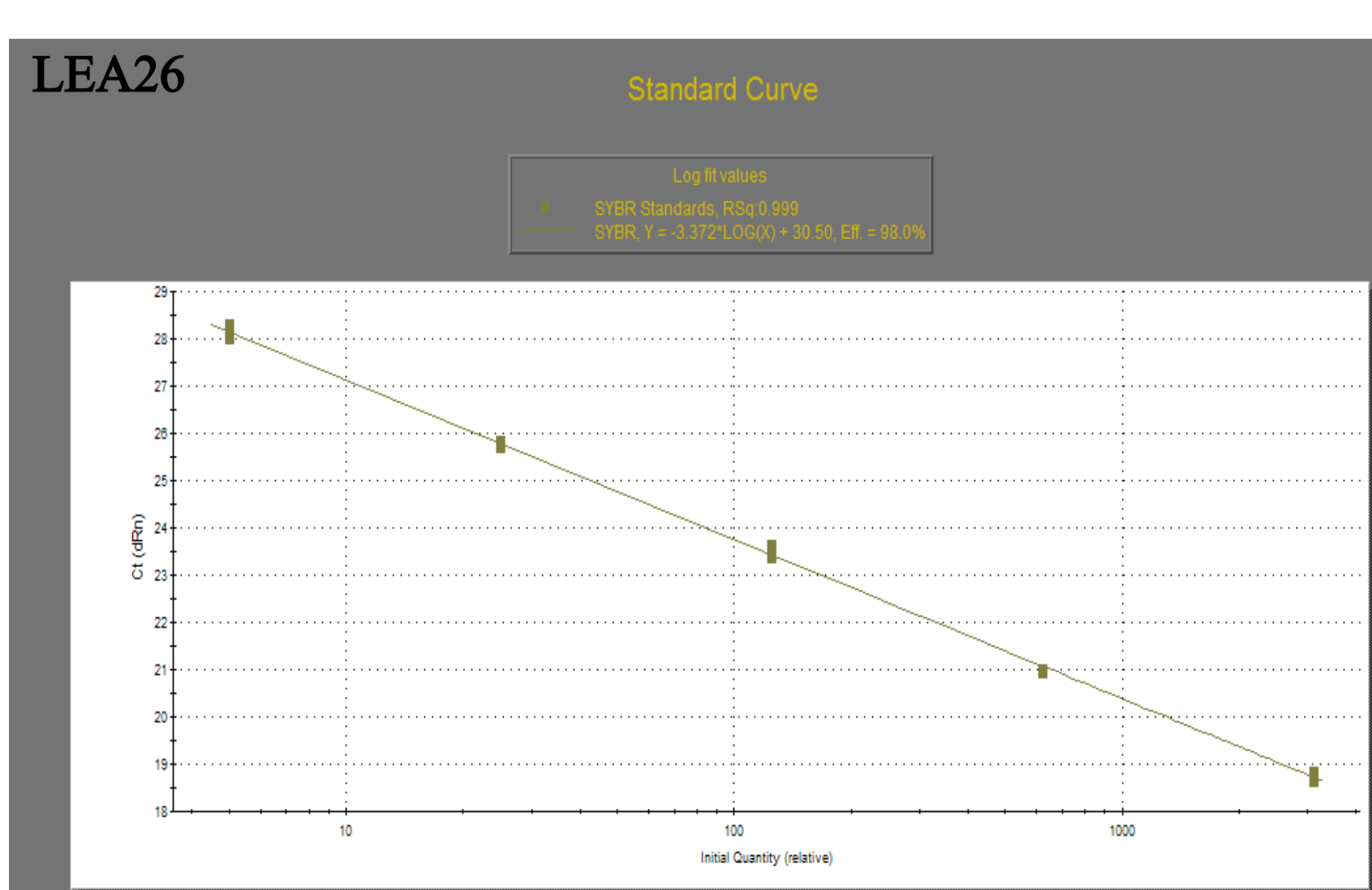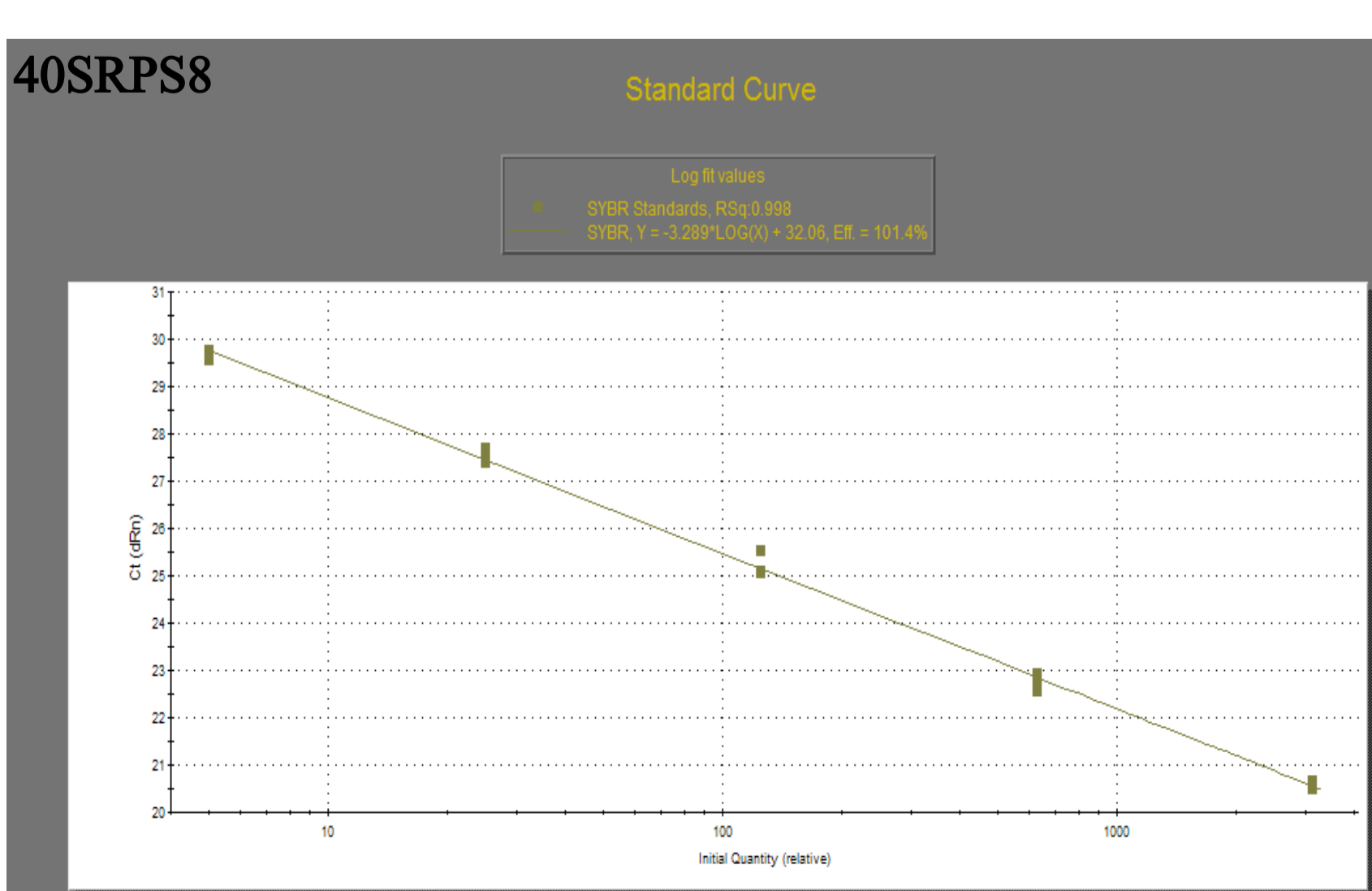

Supplement: Figure S4 — The Fluorescence changes (Y-axis) was plotted versus the reaction temperature of qRT-PCR (X-axis). A single peak of the melting curve in qRT-PCR were used to ensure the specificity of the primers for the candidate reference genes (Fig. S4A).The amplification efficiencies for each primer and the regression coefficients (R2) were evaluated using five-fold dilutions of pooled cDNA (1/5, 1/25, 1/125, 1/625, 1/3125) that were diluted using EASY dilution solution, the Ct values changes (Y-axis) was plotted versus the initial quality of qRT-PCR (X-axis). The linear regression equation of every primer was also showed in Fig. S4B. [file peerj-07-6536-s009.pdf]

A

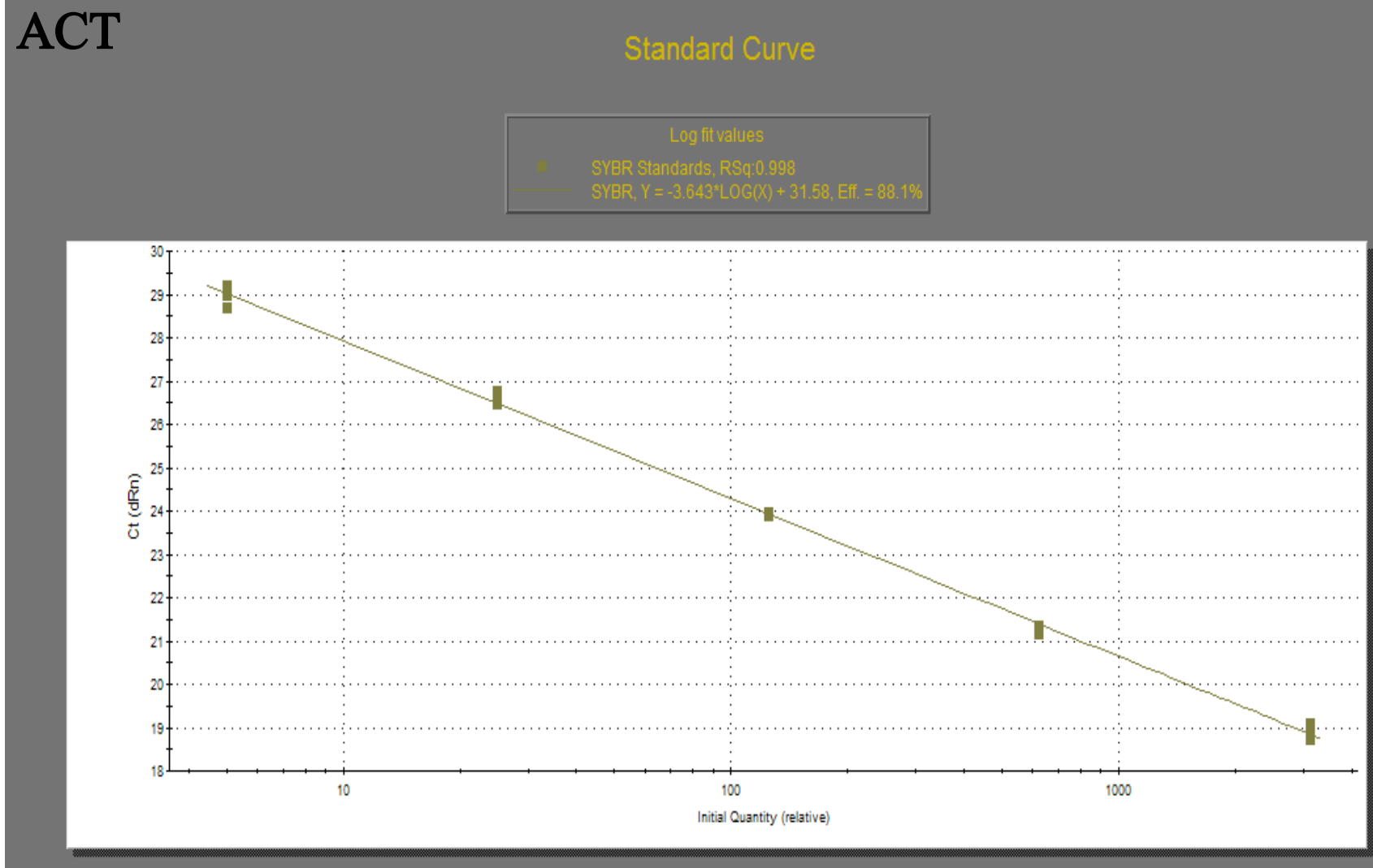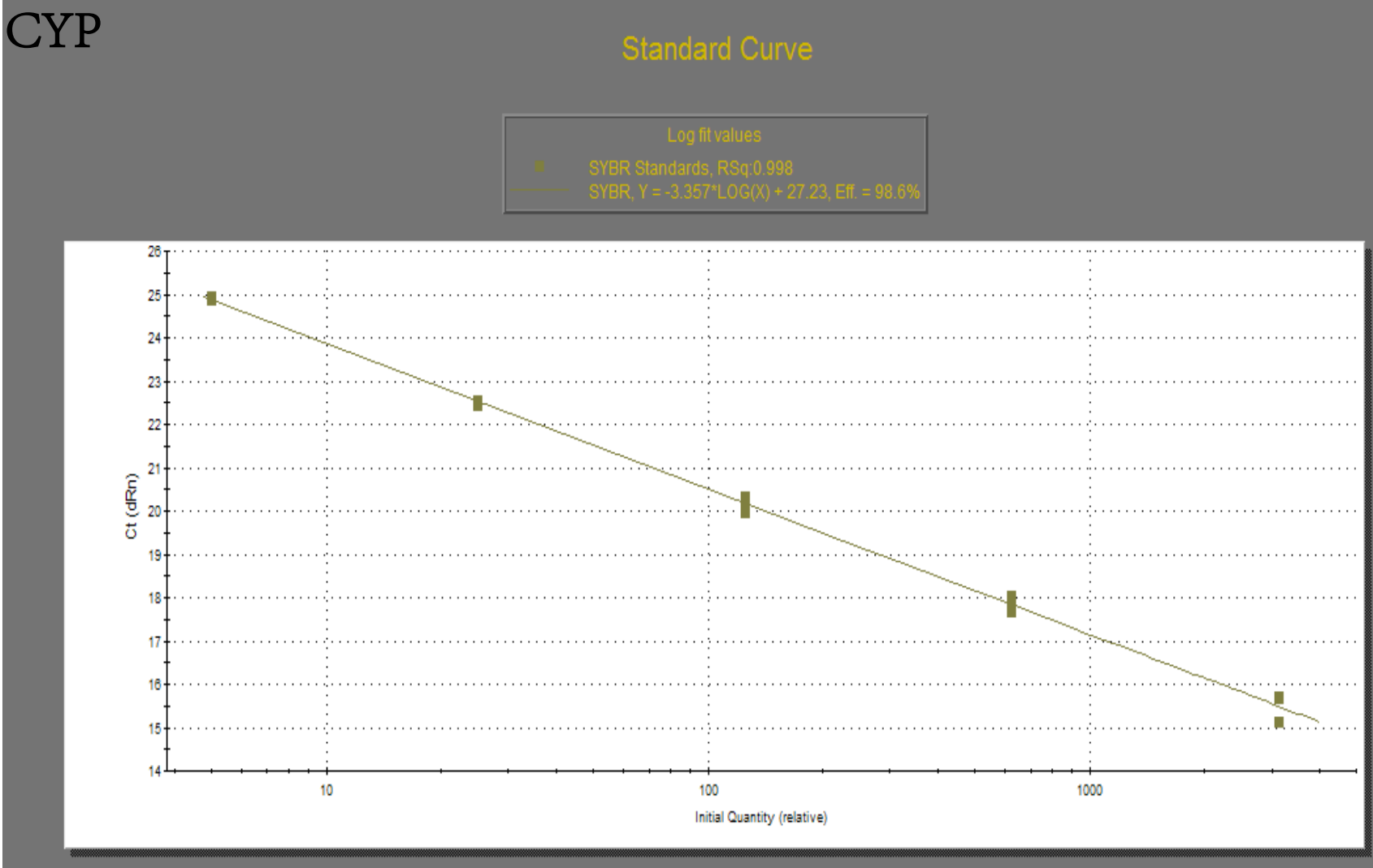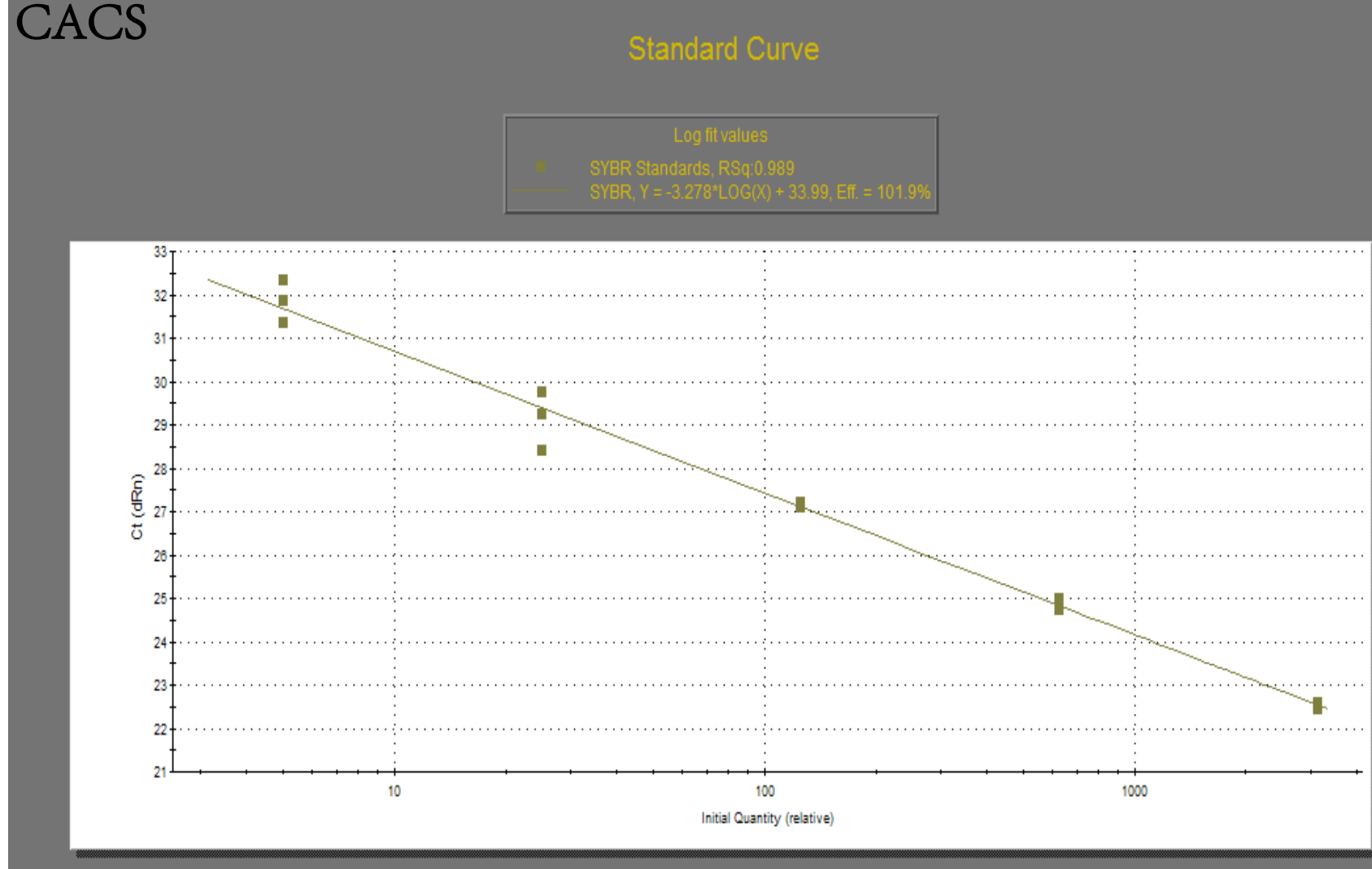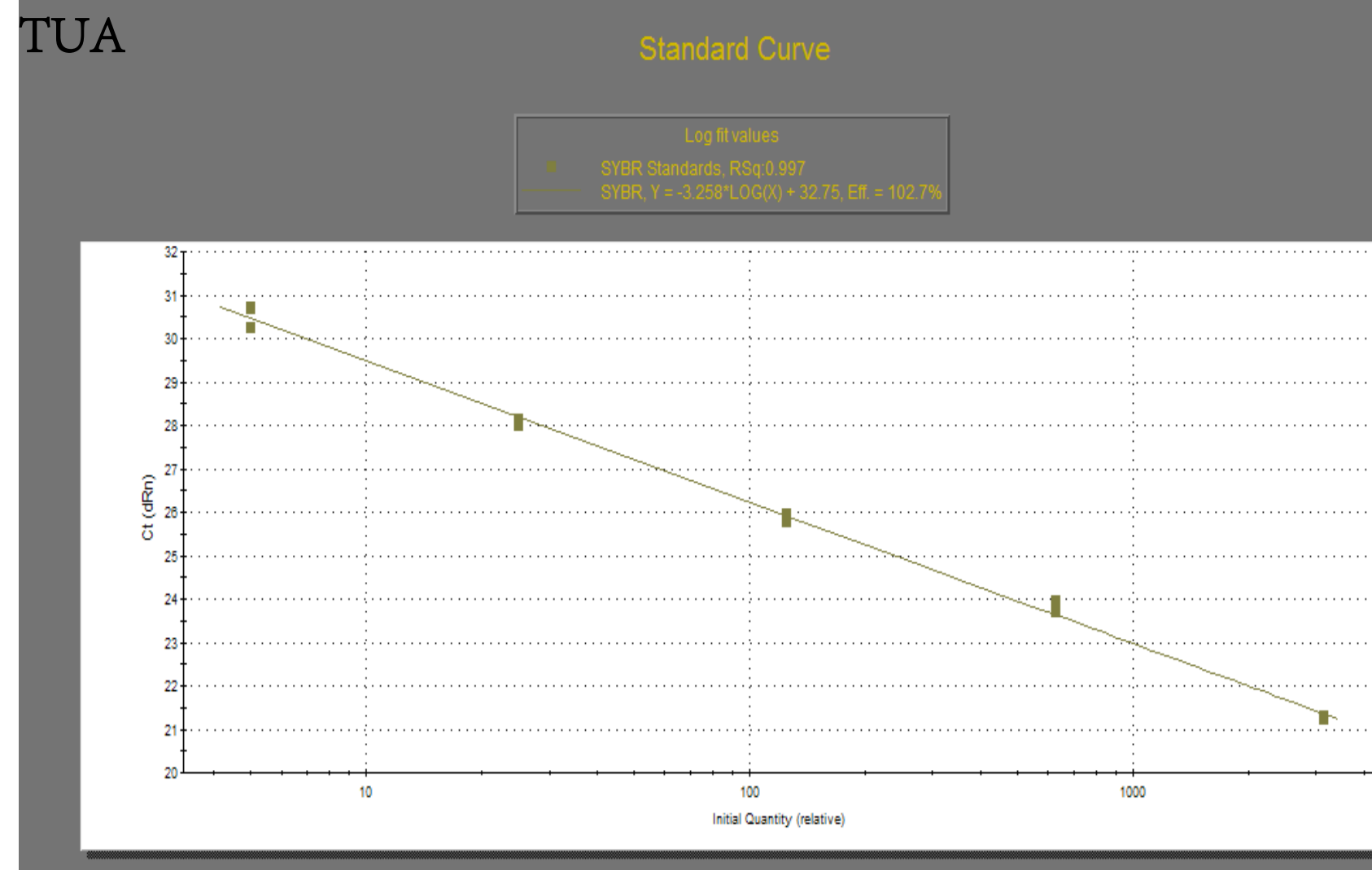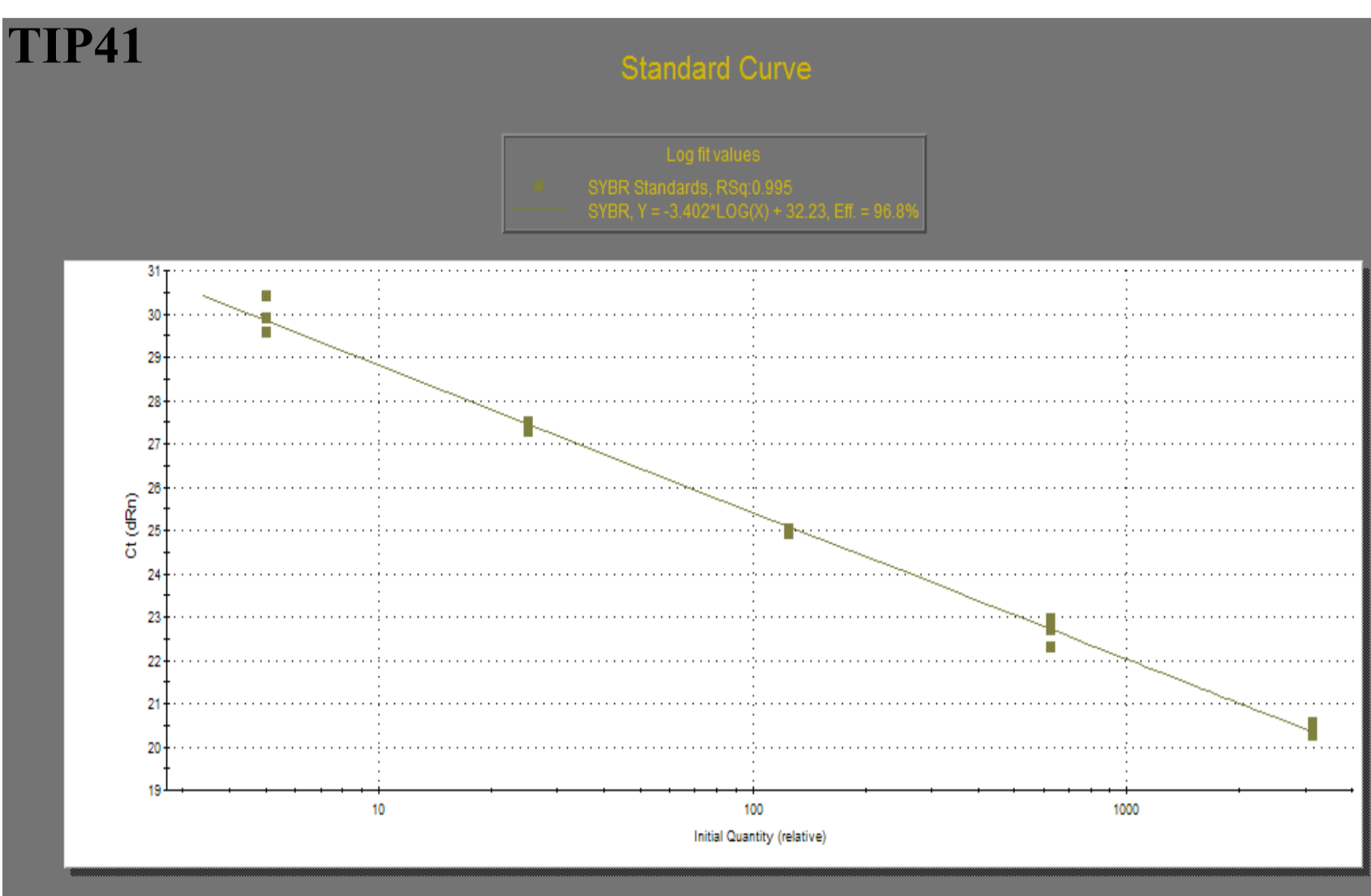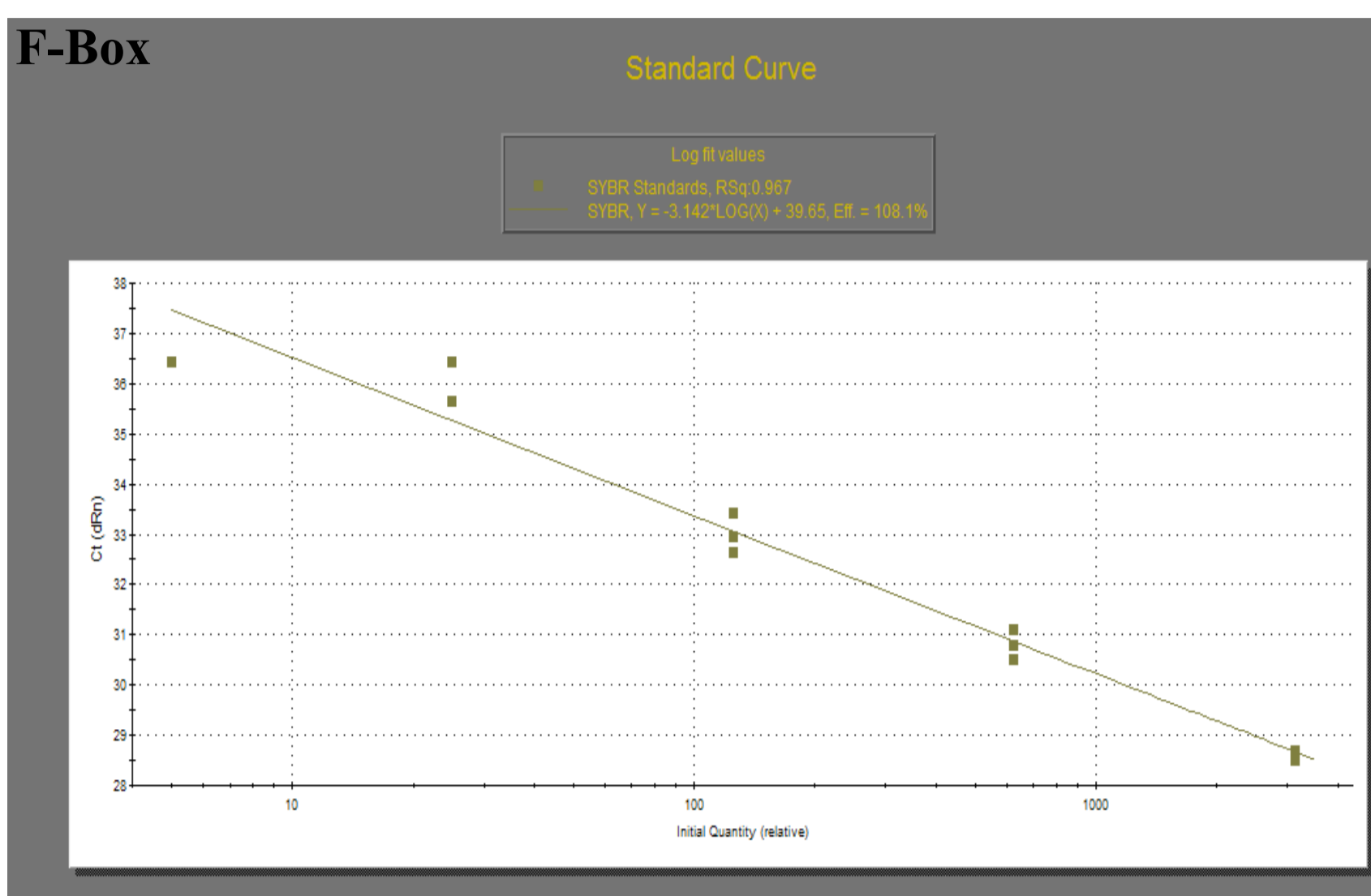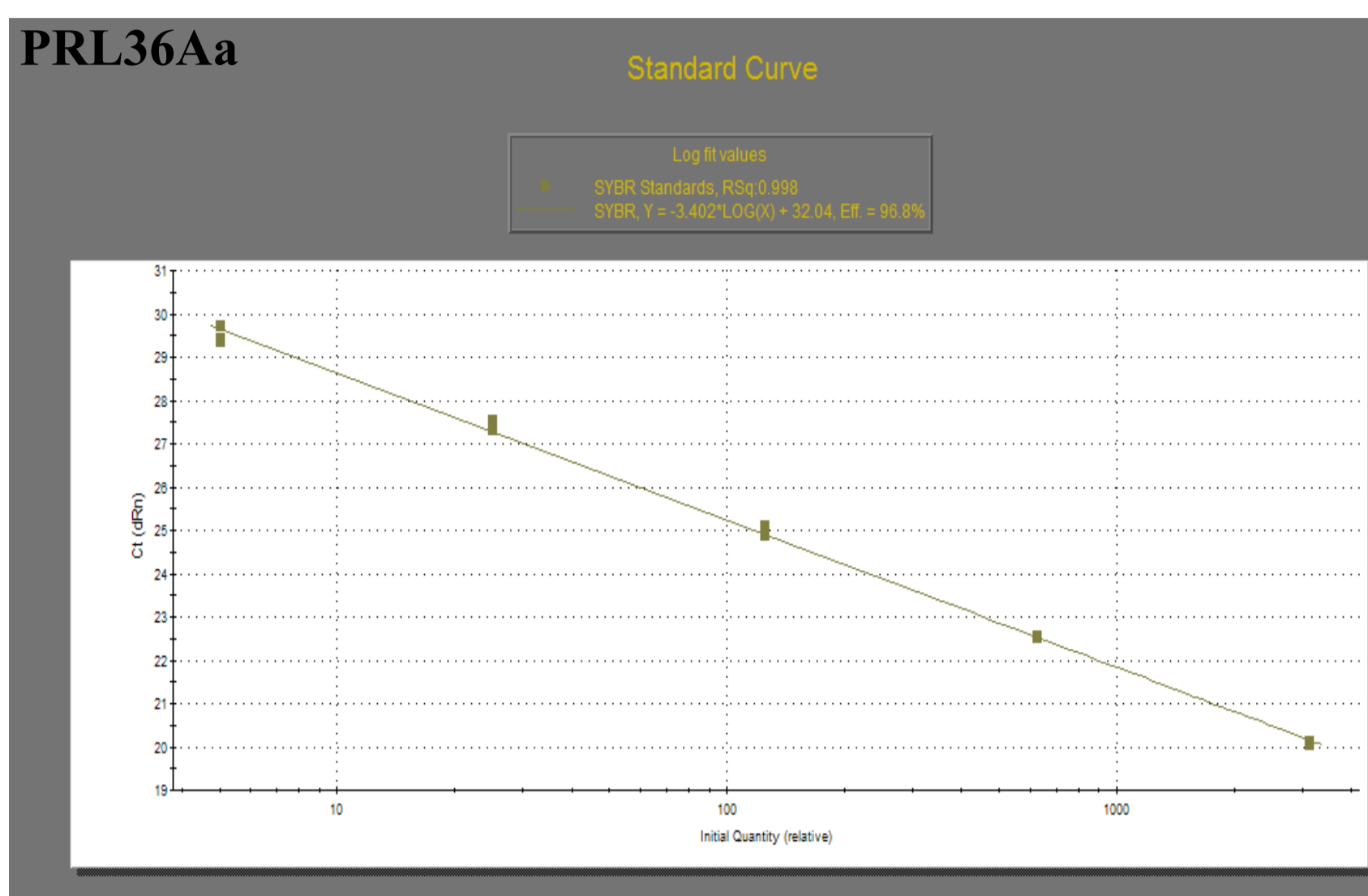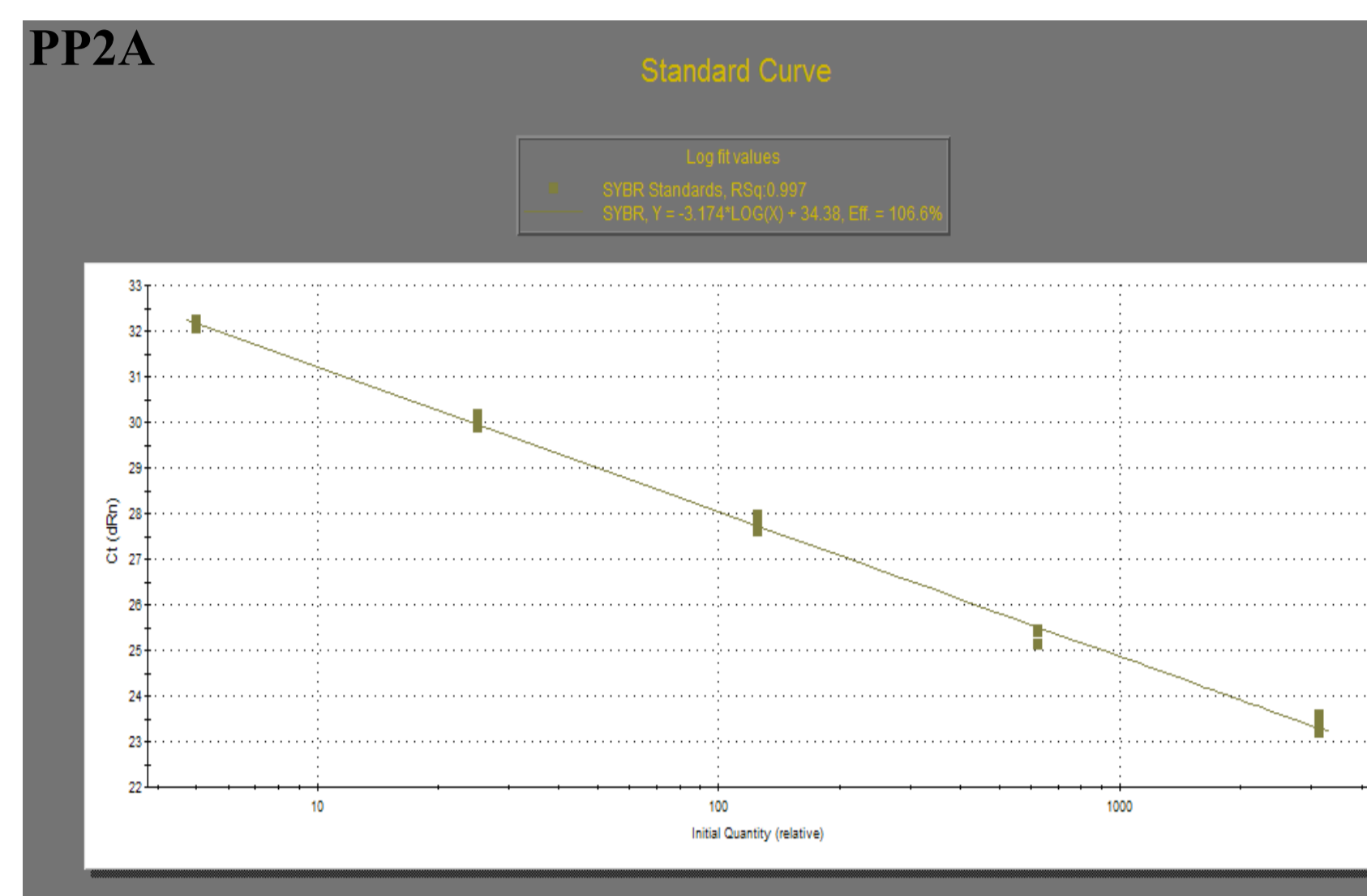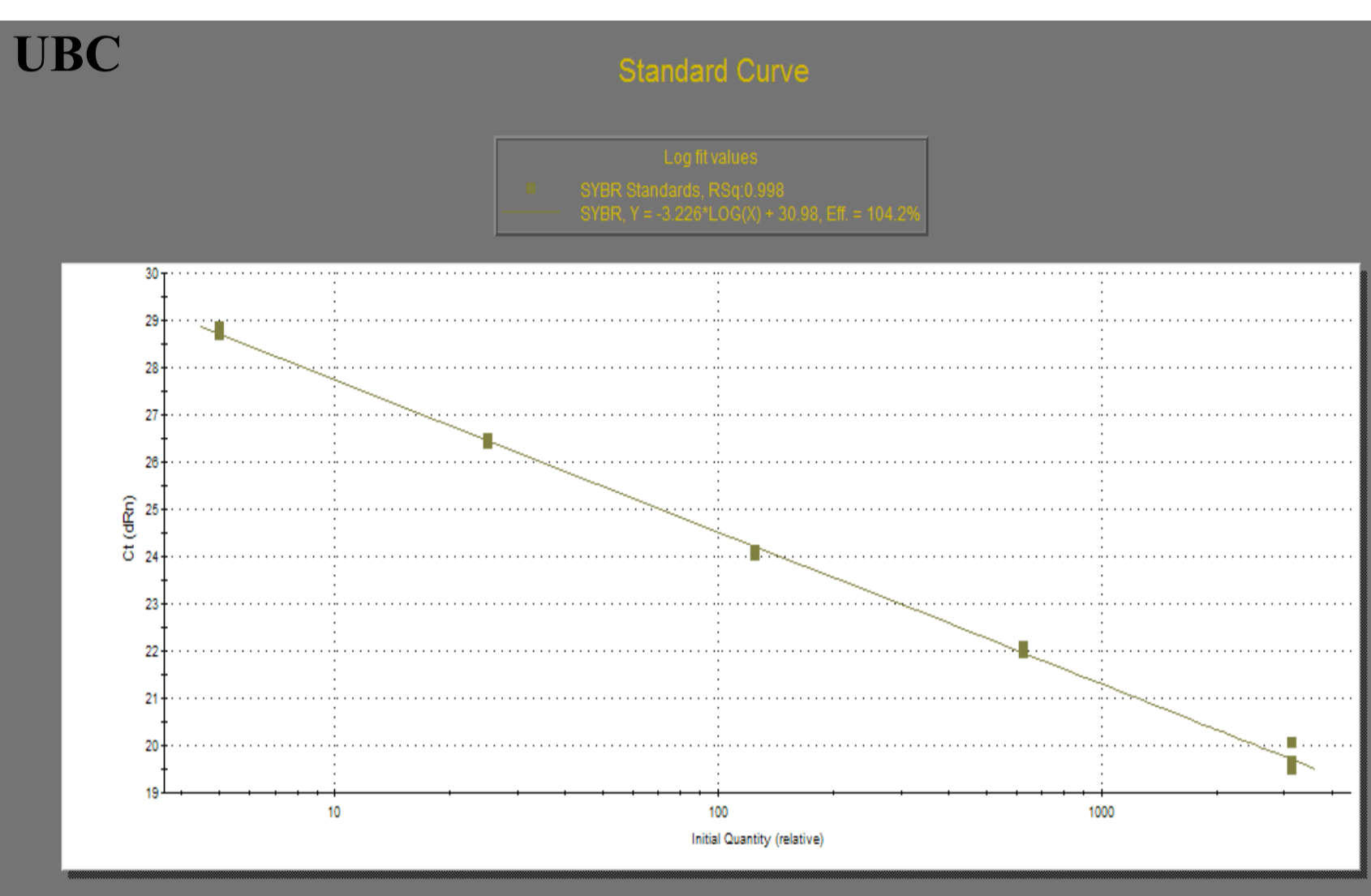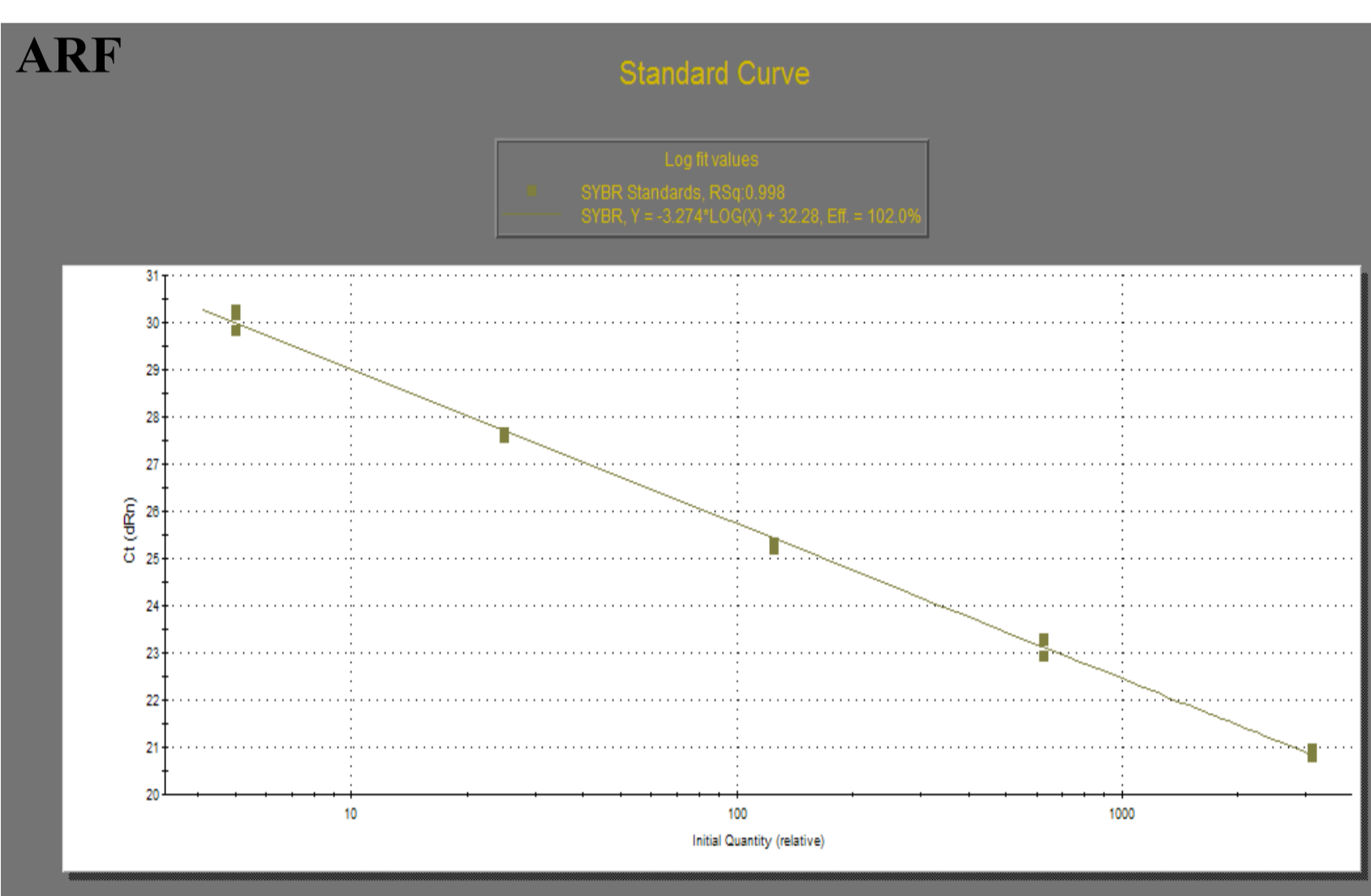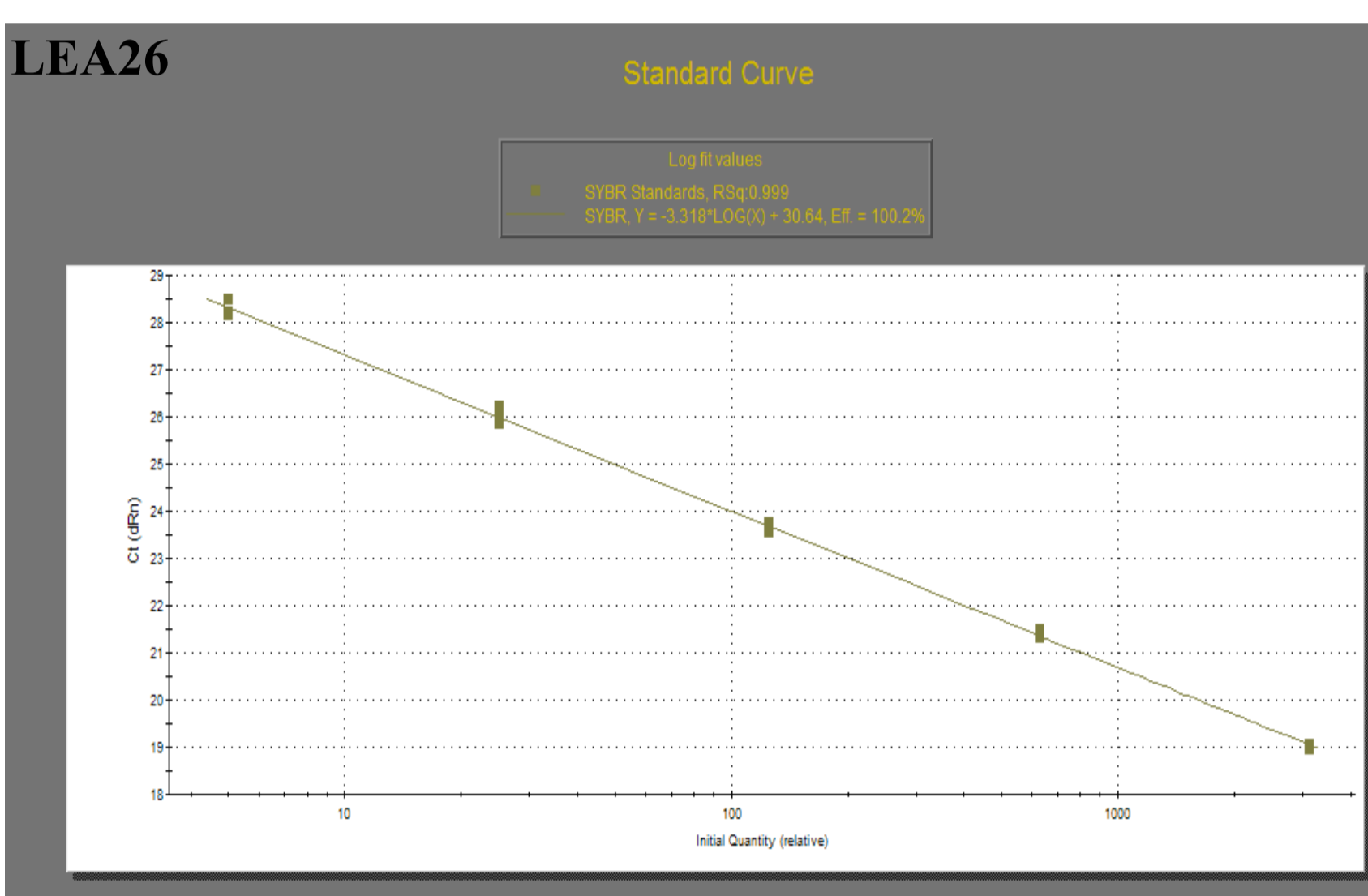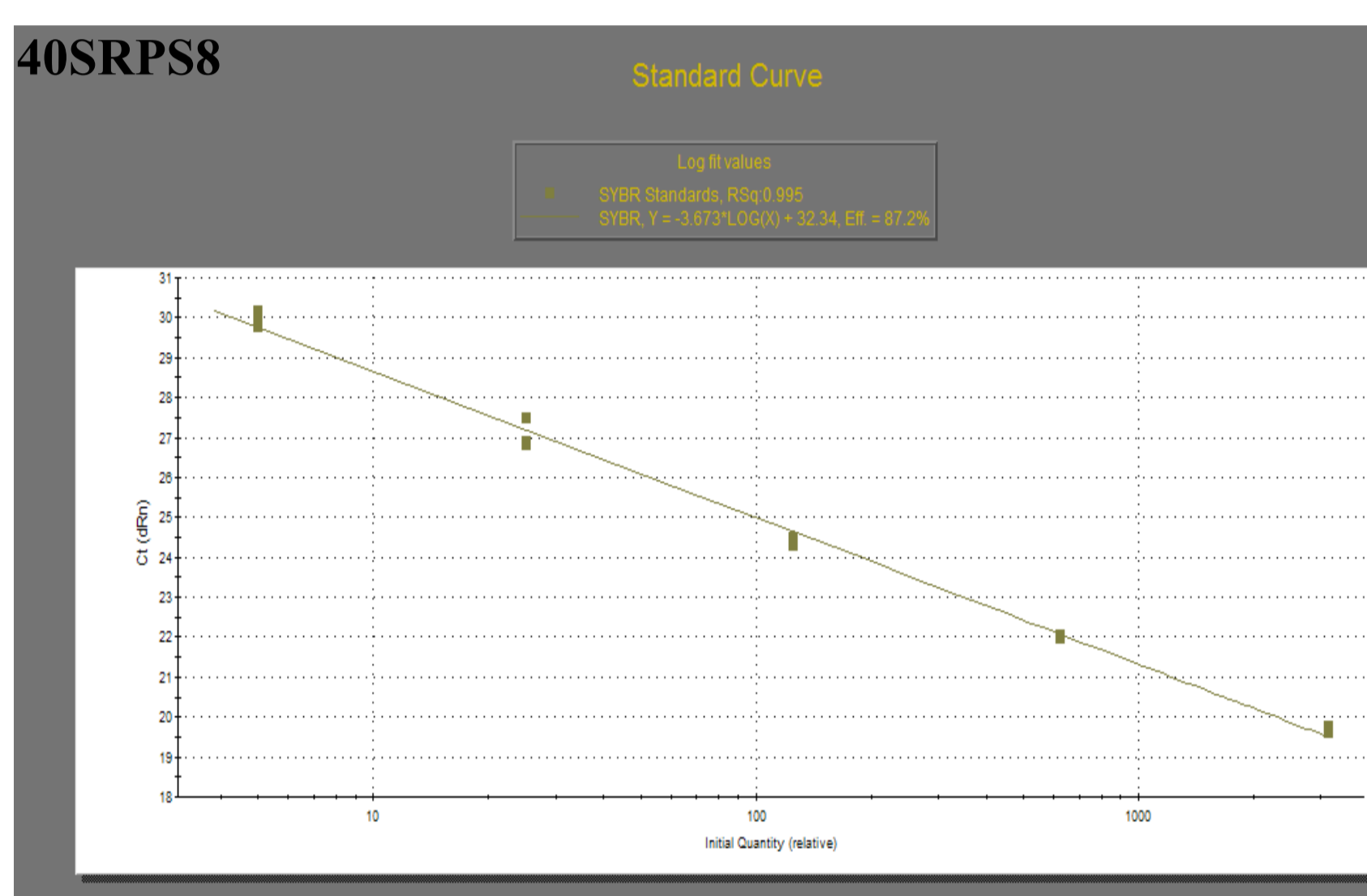

B

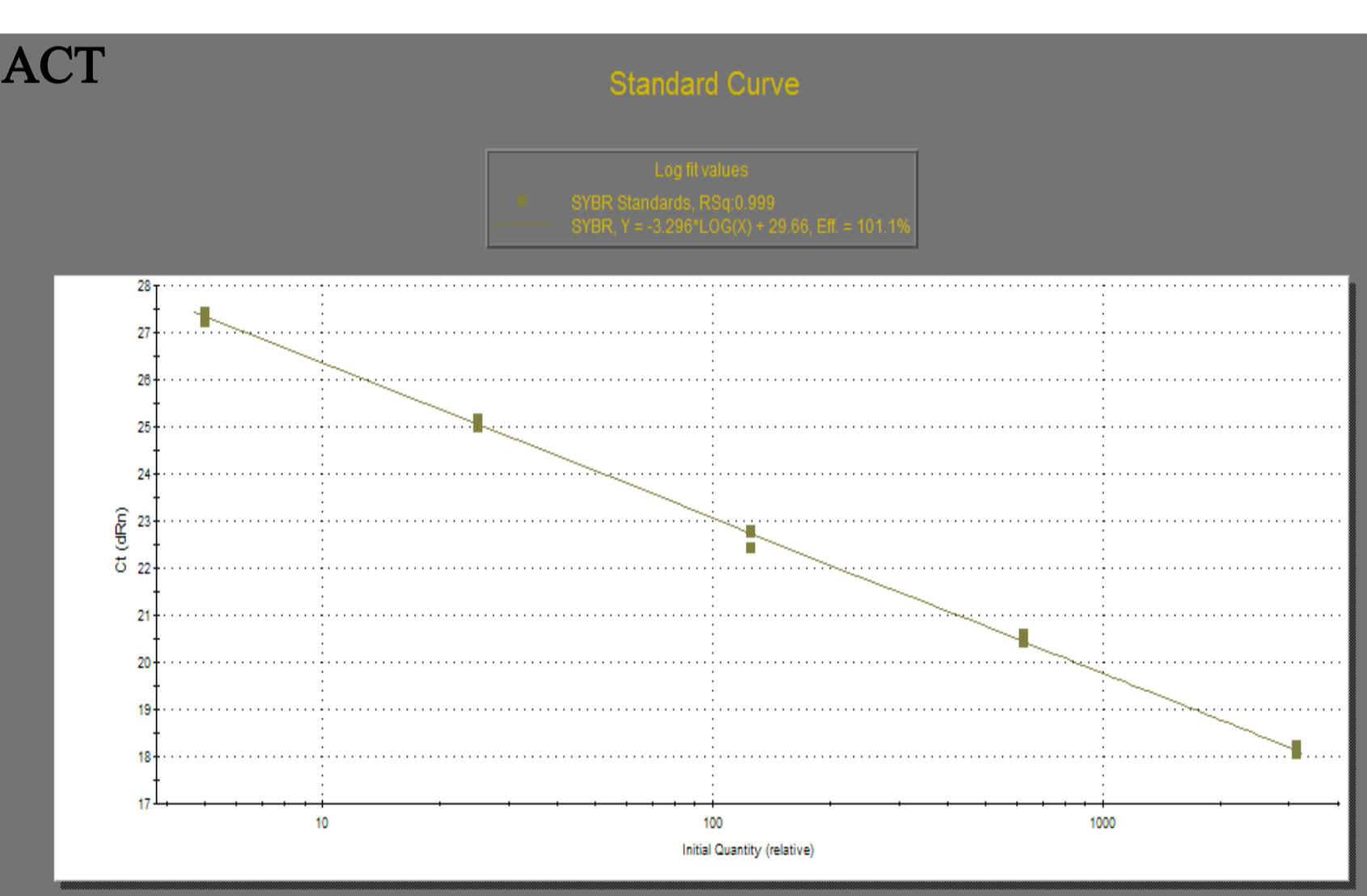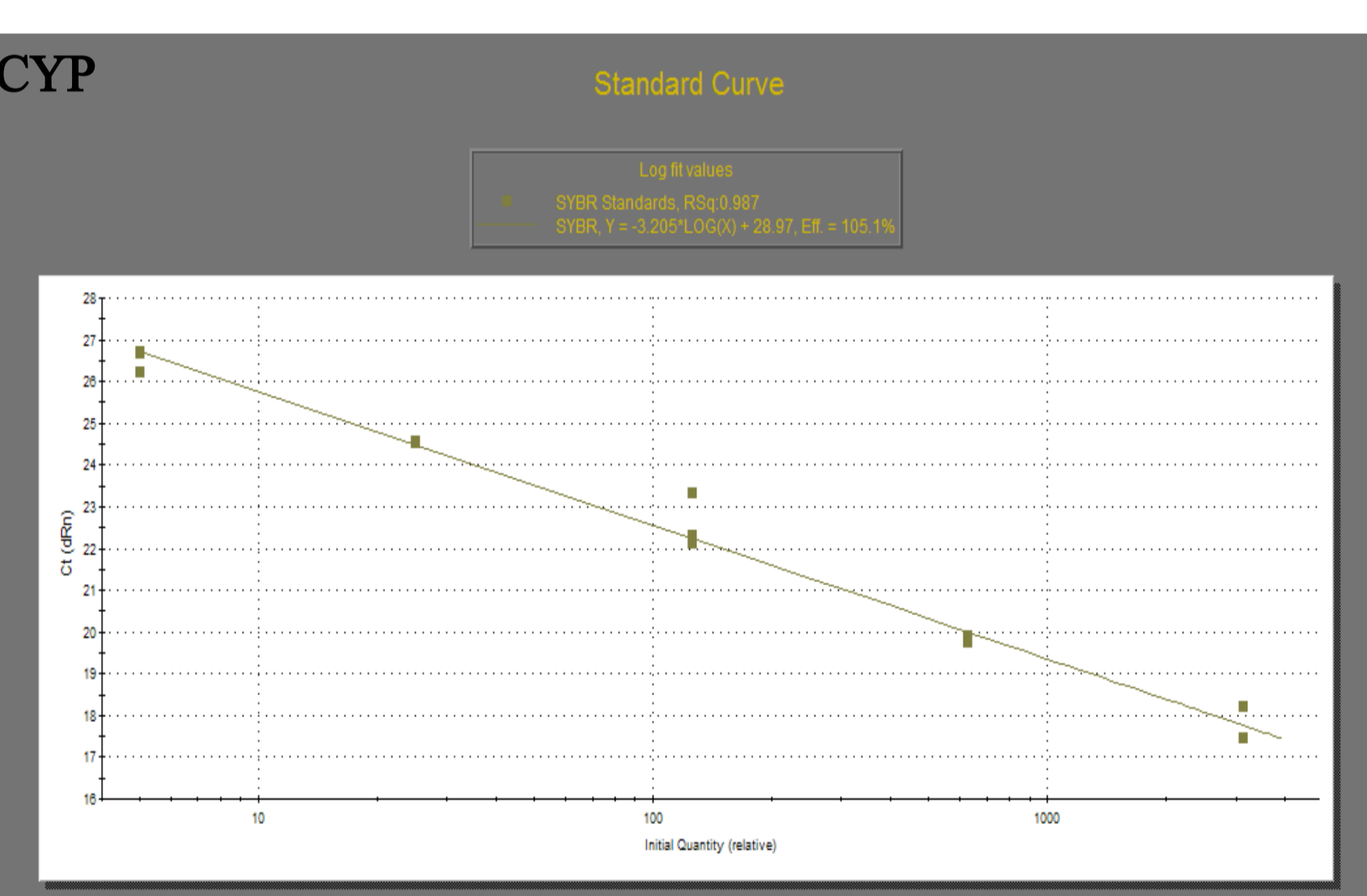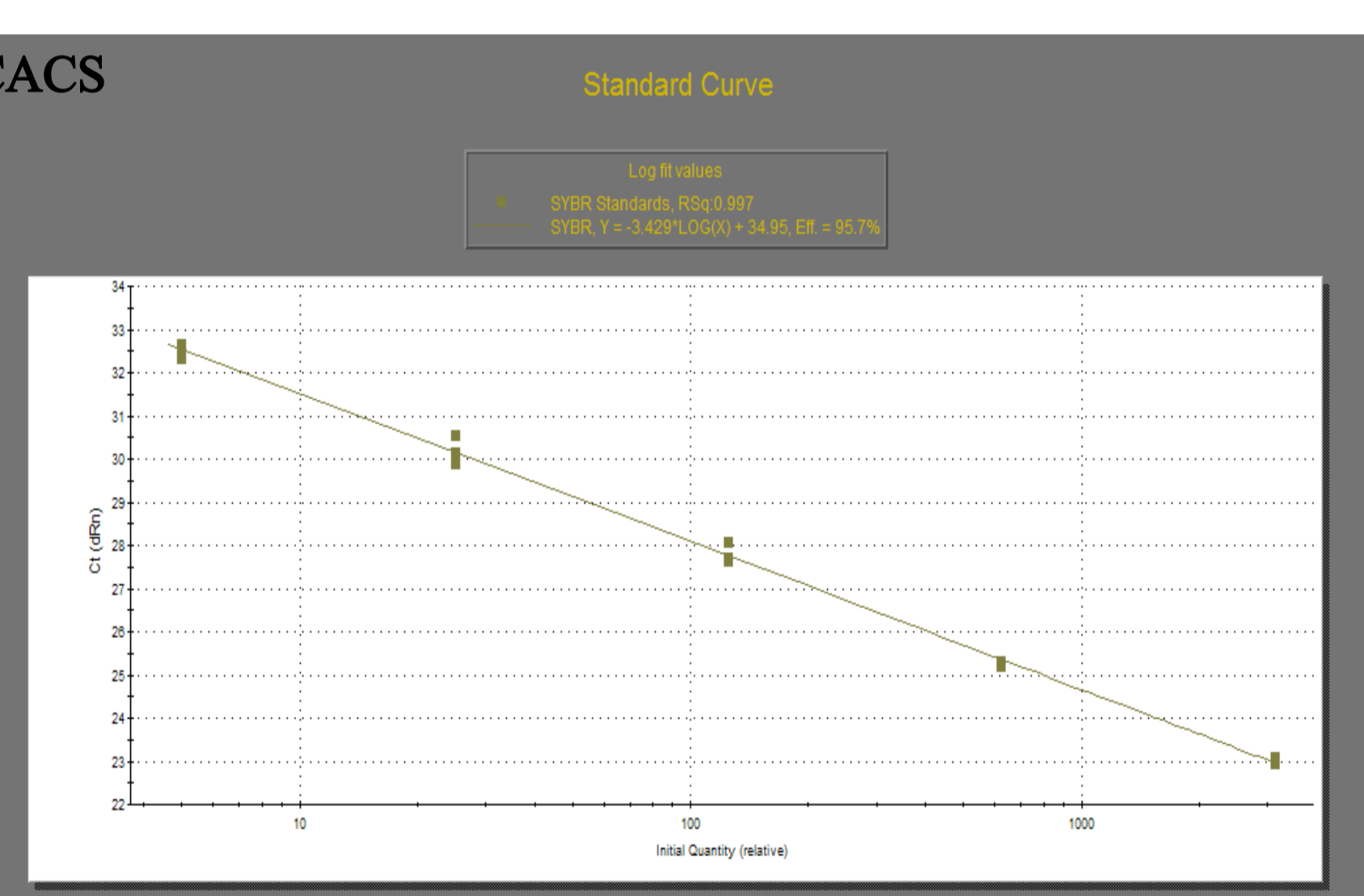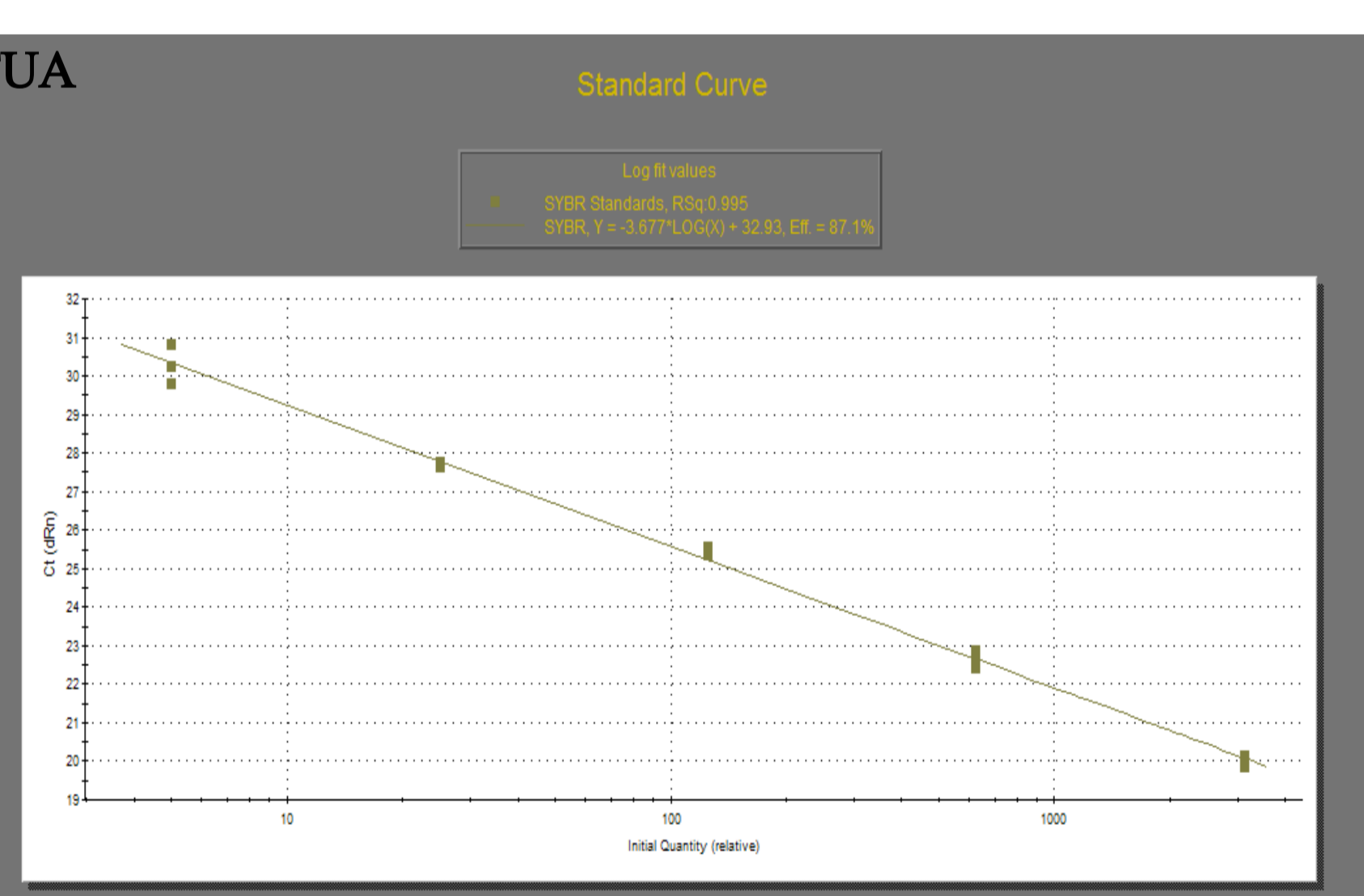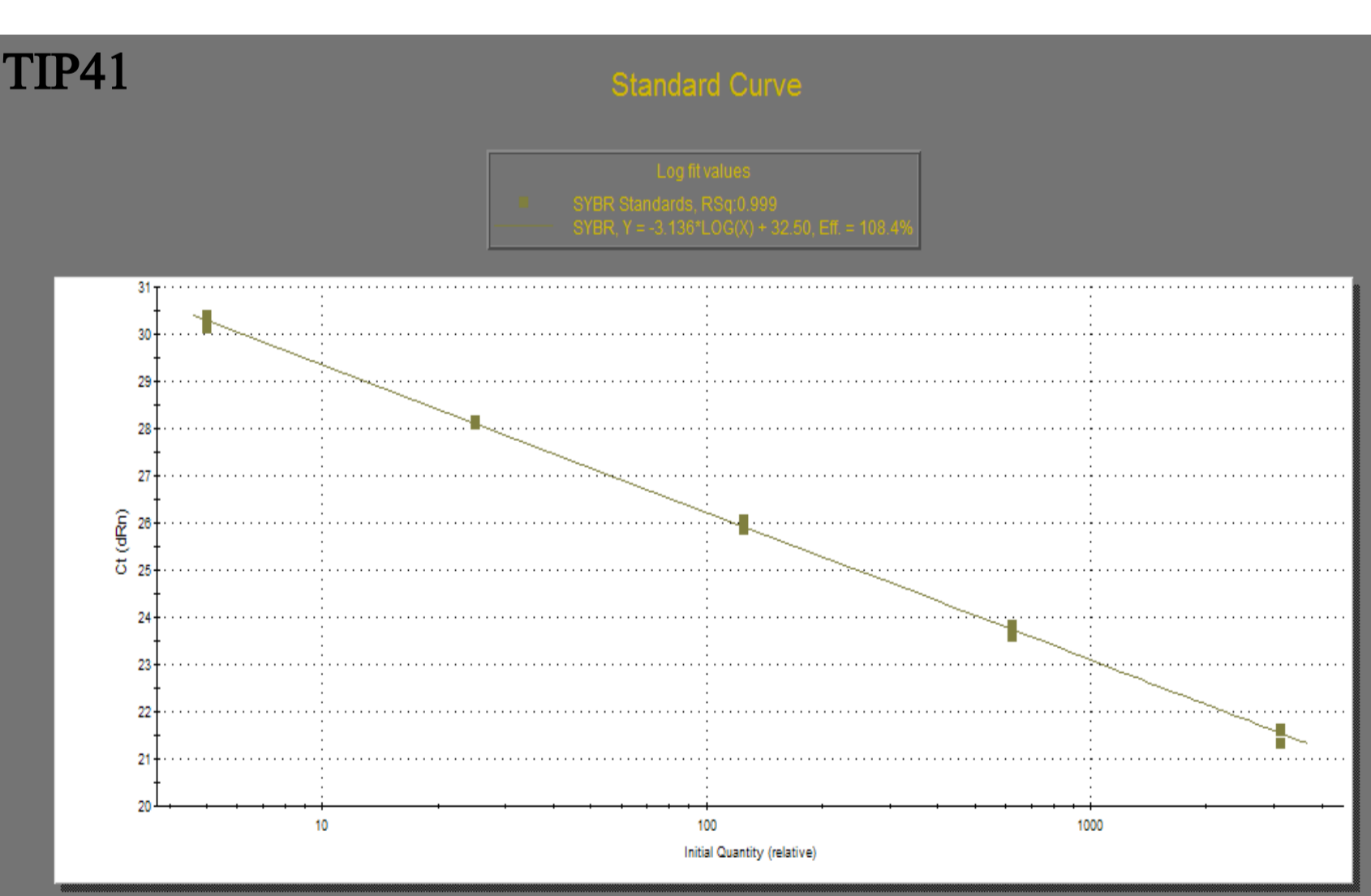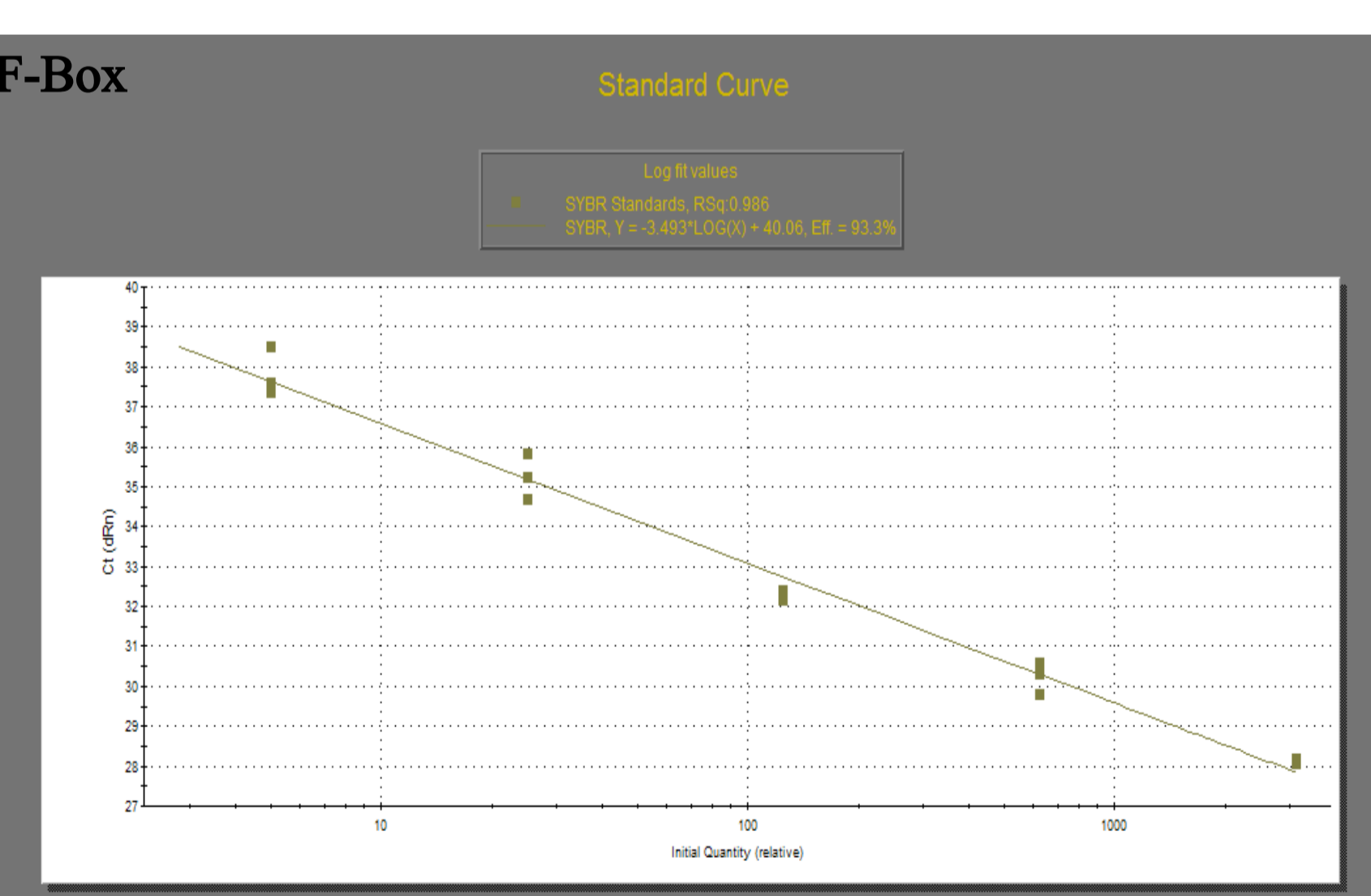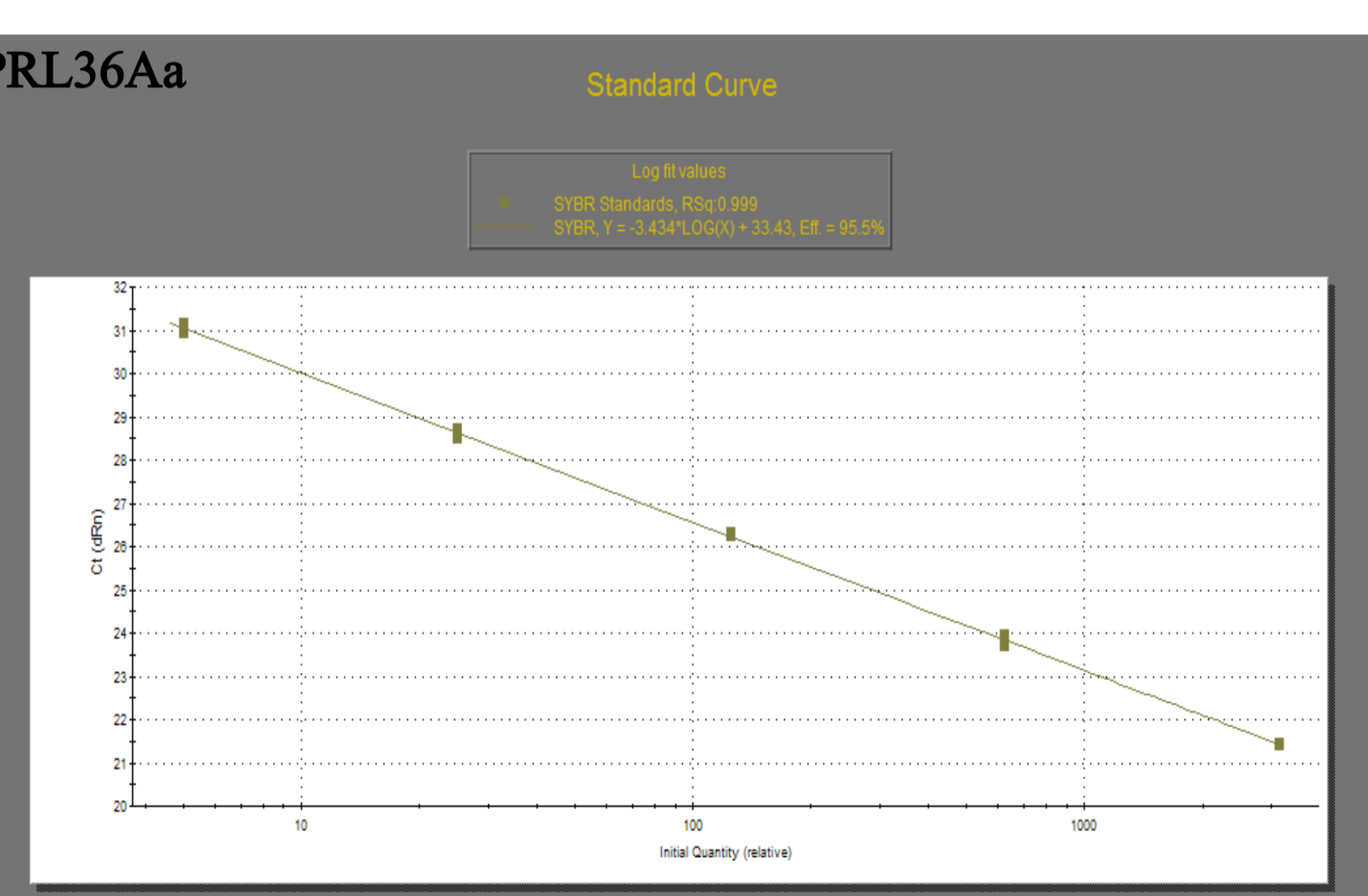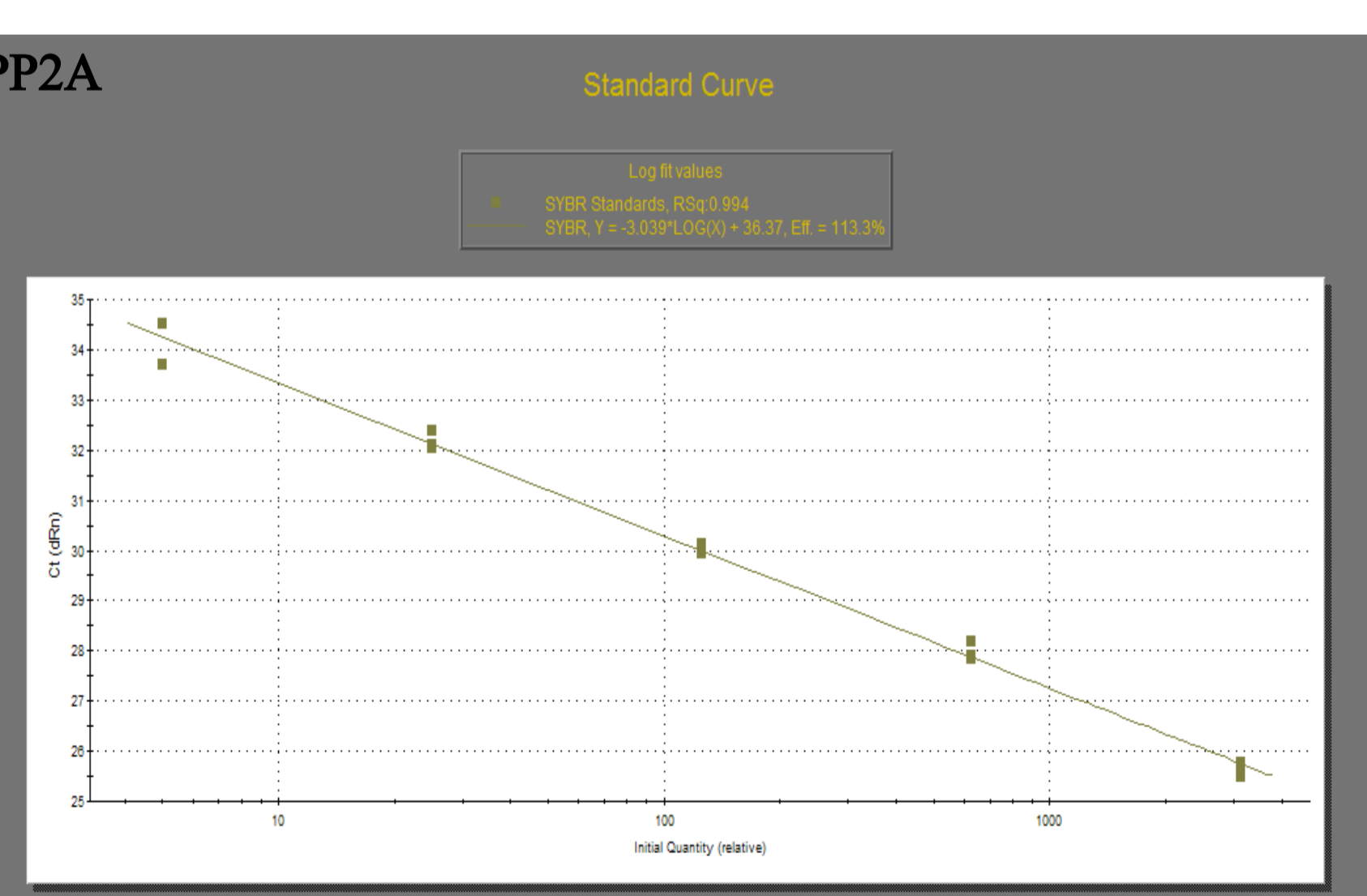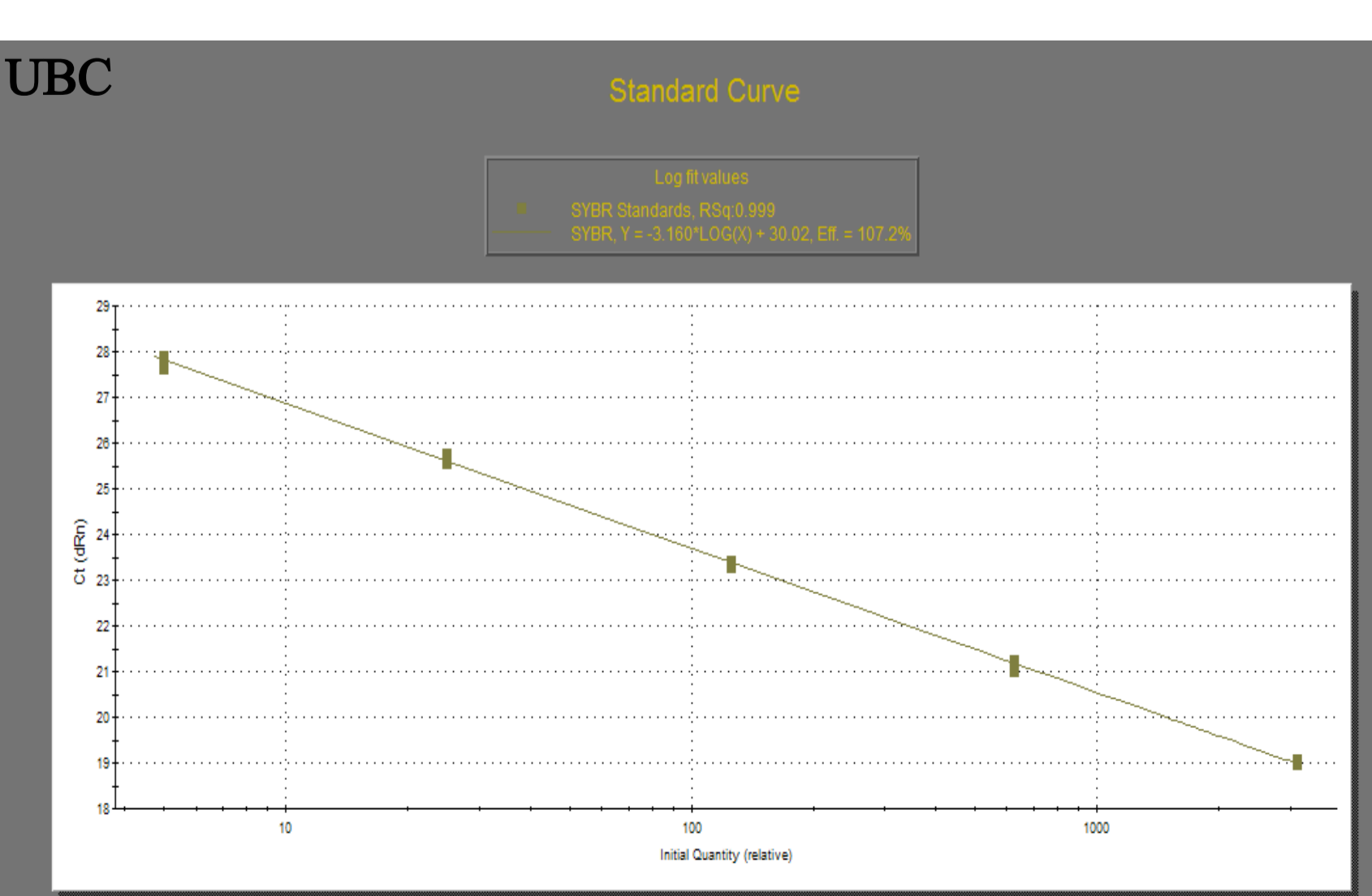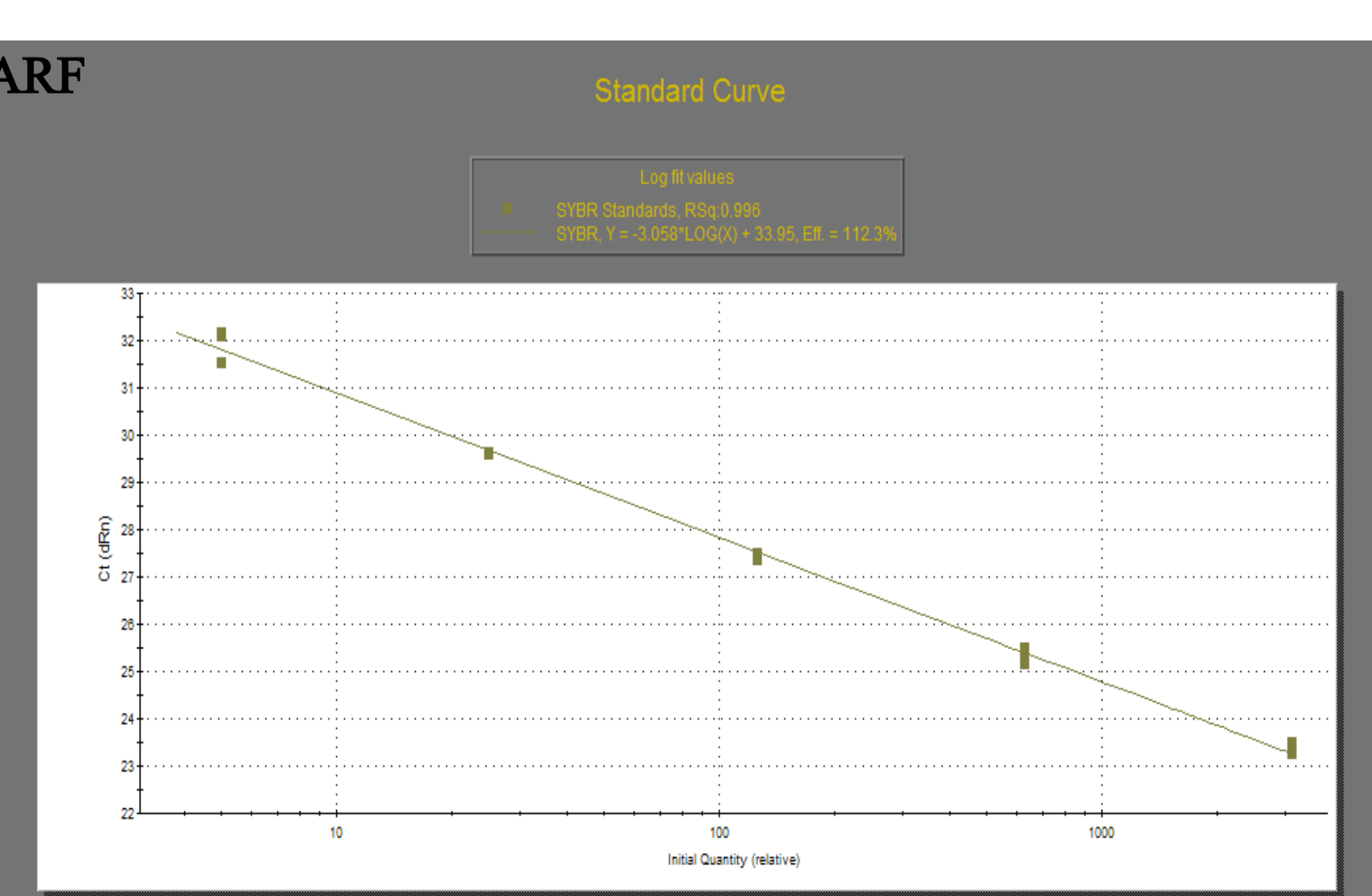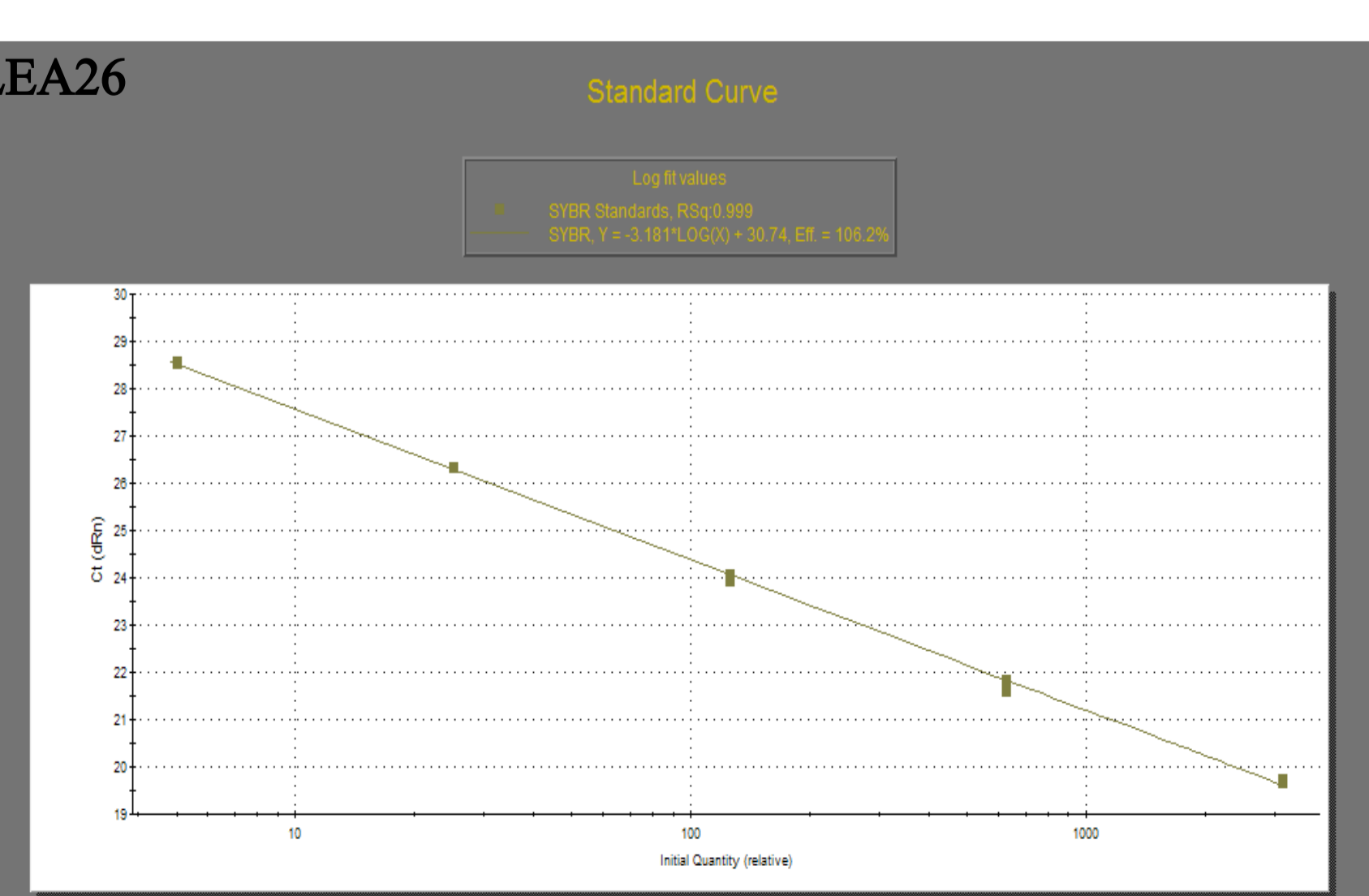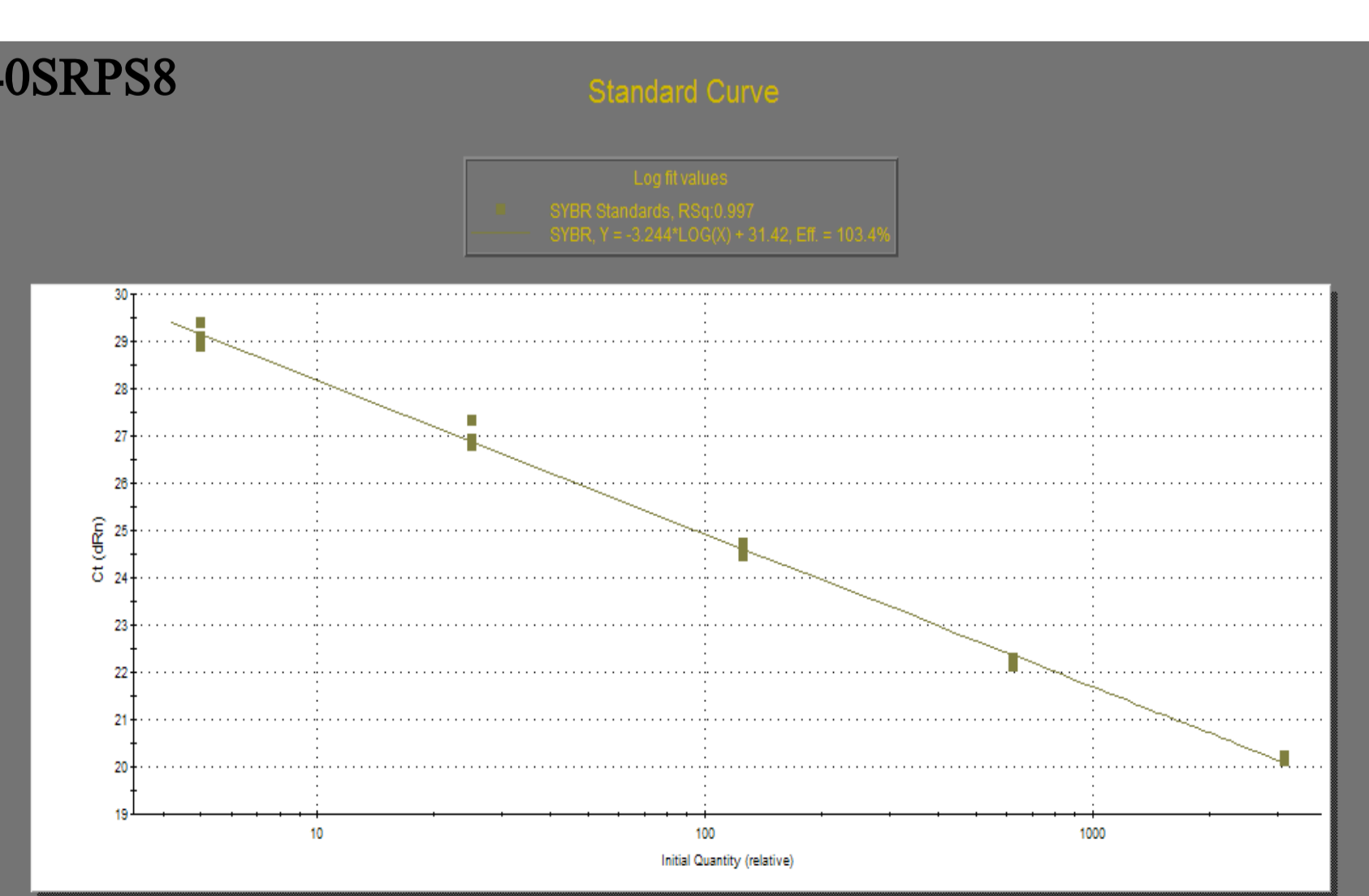

Supplement: Figure S5 — The amplification efficiencies for each primer and the regression coefficients (R2) were evaluated using five-fold dilutions of pooled cDNA (1/5, 1/25, 1/125, 1/625, 1/3125) that were diluted using EASY dilution solution. The Ct values changes (Y-axis) was plotted versus the initial quality of qRT-PCR (X-axis. The linear regression equation of every primer was also showed in Fig. S5. [file peerj-07-6536-s010.pdf]
